# Toxicoproteomic Profiling of *hPXR* Transgenic Mice Treated with Rifampicin and Isoniazid

**DOI:** 10.3390/cells9071654

**Published:** 2020-07-09

**Authors:** Christopher Trent Brewer, Kiran Kodali, Jing Wu, Timothy I. Shaw, Junmin Peng, Taosheng Chen

**Affiliations:** 1 Department of Chemical Biology and Therapeutics, St. Jude Children’s Research Hospital, Memphis, TN 38105, USA; cbrewe16@uthsc.edu (C.T.B.); jing.wu@stjude.org (J.W.); 2 College of Medicine, University of Tennessee Health Science Center, Memphis, TN 38163, USA; 3 Integrated Biomedical Sciences Program, College of Graduate Health Sciences, University of Tennessee Health Science Center, Memphis, TN 38163, USA; 4 Center for Proteomics and Metabolomics, St. Jude Children’s Research Hospital, Memphis, TN 38105, USA; kiran.kodali@stjude.org (K.K.); tim.shaw@stjude.org (T.I.S.); 5 Department of Computational Biology, St. Jude Children’s Research Hospital, Memphis, TN 38105, USA; 6 Department of Structural Biology, St. Jude Children’s Research Hospital, Memphis, TN 38105, USA; 7 Department of Developmental Neurobiology, St. Jude Children’s Research Hospital, Memphis, TN 38105, USA

**Keywords:** anemia, antitubercular therapy, cytochrome P450, drug-induced liver injury, heme biosynthesis, hypercoagulability, iron–sulfur cluster, pellagra, vitamin B_3_, vitamin B_6_

## Abstract

Tuberculosis is a global health threat that affects millions of people every year, and treatment-limiting toxicity remains a considerable source of treatment failure. Recent reports have characterized the nature of *hPXR*-mediated hepatotoxicity and the systemic toxicity of antitubercular drugs. The antitubercular drug isoniazid plays a role in such pathologic states as acute intermittent porphyria, anemia, hepatotoxicity, hypercoagulable states (deep vein thrombosis, pulmonary embolism, or ischemic stroke), pellagra (vitamin B_3_ deficiency), peripheral neuropathy, and vitamin B_6_ deficiency. However, the mechanisms by which isoniazid administration leads to these states are unclear. To elucidate the mechanism of rifampicin- and isoniazid-induced liver and systemic injury, we performed tandem mass tag mass spectrometry-based proteomic screening of *mPxr*^−/−^ and *hPXR* mice treated with combinations of rifampicin and isoniazid. Proteomic profiling analysis suggested that the *hPXR* liver proteome is affected by antitubercular therapy to disrupt [Fe–S] cluster assembly machinery, [2Fe–2S] cluster-containing proteins, cytochrome P450 enzymes, heme biosynthesis, homocysteine catabolism, oxidative stress responses, vitamin B_3_ metabolism, and vitamin B_6_ metabolism. These novel findings provide insight into the etiology of some of these processes and potential targets for subsequent investigations. Data are available via ProteomeXchange with identifier PXD019505.

## 1. Introduction

Tuberculosis (TB) is a leading cause of death from infectious diseases worldwide, second only to human immunodeficiency virus [1]. The treatment for latent TB infections comprises isoniazid treatment for 6 months. Treatment for active TB infections comprises 4 months of rifampicin, isoniazid, pyrazinamide, and ethambutol. Hepatotoxicity decreases adherence to antitubercular treatment regimens and contributes to treatment failure, disease relapse, and drug resistance [2]. Hepatotoxicity is a leading cause of TB therapy treatment failure, with up to 28% of all anti-TB treatment failure attributed to toxicity [3,4]. Antitubercular therapy may also be associated with acute intermittent porphyria [5,6], sideroblastic anemia [7,8,9,10], hypercoagulability [11,12,13], peripheral neuropathy [14,15,16], porphyria cutanea tarda [17], vitamin B_3_ deficiency [18,19,20], and vitamin B_6_ deficiency [8,14,21,22].

Further characterization of the mechanism of antitubercular liver and systemic injury is necessary to update clinical guidance to prevent or ameliorate injury. The human pregnane X receptor (hPXR) is implicated in the hepatotoxicity of pharmaceuticals [23,24] and herbal supplements [25]. This hepatotoxicity is associated with increased protoporphyrin IX (PPIX), which is a precursor to heme suitable for quantification via high-throughput fluorescence imaging [26]. The iron-dependent modulation of ferrochelatase (FECH) and aminolevulinic acid synthase 1 (ALAS1) is also driven by metabolites of isoniazid [27]. Rifampicin is a well-known inducer of drug-metabolizing enzymes and transporters via hPXR. The hPXR is a nuclear hormone receptor that responds to endobiotics and xenobiotics by increasing transcription of the genes involved in the elimination of toxic compounds, such as cytochrome P450 (CYP) enzymes, glucuronosyltransferases, sulfotransferases, ATP-binding cassette transports, and others. Isoniazid is a vitamin B_6_ antimetabolite that is associated with clinical syndromes exacerbated by and associated with vitamin B_6_ depletion.

Isoniazid and rifampicin treatment alone and in combination can lead to drug toxicity. The toxicity of the combination may be more severe and/or frequent than either drug alone. In mice, isoniazid- and rifampicin-induced hepatotoxicity is hPXR-dependent and associated with accumulation of PPIX. These antitubercular drugs are administered orally and undergo first-pass metabolism in the liver. Consequently, many of the processes involved in the pathophysiology of systemic antitubercular toxicity are located in the liver.

We investigated the effects of the antitubercular drugs rifampicin and isoniazid by tandem mass tag (TMT) mass spectrometry-based proteomic screening of the livers from mice with *Pxr* knockout (*mPxr*^−/−^) and the *mPxr*^−/−^ mice with transgenic *hPXR* (*hPXR* mice) treated with rifampicin, isoniazid, or a combination of rifampicin and isoniazid. We specifically used *hPXR* transgenic mice because rifampicin is a specific agonist for hPXR and not mouse pregnane X receptor (PXR) [28]. Proteomic profiling identified changes in [Fe–S] cluster assembly machinery proteins, [Fe–S] cluster-containing proteins, CYP and steroid metabolism enzymes, heme synthesis and degradation, iron metabolism, nuclear hormone receptors, oxidative stress, tryptophan metabolism, vitamin B_3_ metabolism, vitamin B_6_ metabolism, and wound-healing and inflammation pathways. This study is the first to investigate the role of hPXR in these pathways by using both rifampicin and isoniazid in *hPXR* mice (compared to *mPxr*^−/−^mice) to identify hPXR-mediated changes in protein levels.

## 2. Materials and Methods

### 2.1. Reagents

We obtained dimethyl sulfoxide from Corning (Corning, NY, USA), Dulbecco phosphate-buffered saline (PBS) from Gibco (Little Rock, AR, USA), and rifampicin and isoniazid from Sigma-Aldrich (St. Louis, MO, USA). MS-related reagents included LC-MS grade acetonitrile, water, ammonium acetate, ammonium hydroxide, and formic acid from Sigma-Aldrich (St. Louis, MO, USA).

### 2.2. Antibodies

We purchased primary antibodies from the following sources: anti-FECH mouse monoclonal antibody (sc-377377) from Santa Cruz Biotechnology (Dallas, TX, USA; diluted 1:5000), anti-ALAS1 mouse monoclonal antibody (ab54758) from Abcam (Cambridge, UK; diluted 1:2000), and anti-CYP3A4 K03 mouse monoclonal antibody at a dilution of 1:1000 [29,30,31]. We purchased goat anti-mouse infrared dye (IRDye)-conjugated (680 RD and 800 CW) secondary antibodies from LI-COR (Cambridge, UK) and used them at a dilution of 1:10,000 to visualize proteins [27].

### 2.3. Mice

**Figure 1:**
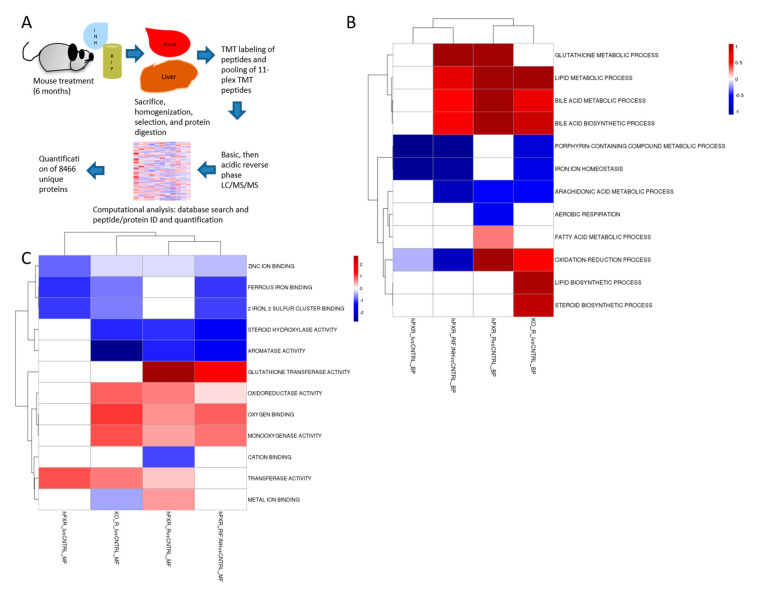
Experimental scheme and gene ontology enrichment analysis. (**A**) Schematic diagram of proteomic analysis workflow: *hPXR* transgenic or *mPxr*^−/−^ mice were treated with rifampicin (RIF, 300 mg/kg chow), isoniazid (INH, 1.2 g/L water), or both. Liver tissues were lysed, digested into peptides, and labeled with tandem mass tags (TMTs). The labeled samples were equally mixed and further fractionated by basic pH reverse-phase liquid chromatography (LC). The fractions were collected and further analyzed by acidic pH reverse-phase LC-MS/MS. During ion fragmentation, the TMT regents were cleaved to produce reporter ions for quantification. The collected data were searched against a database to identify peptides. Although the peptides were identified by MS/MS, quantification was achieved by the fragmented reporter ions in the same MS/MS scans. Then, the peptide quantification data were corrected for mixing errors, summarized to derive protein quantification, and subjected to statistical analysis to determine cutoffs for altered proteins and evaluate the associated false discovery rate. (**B**,**C**) Clustering analysis of the up-regulated and down-regulated proteins in *hPXR* mice treated with rifampicin and/or isoniazid, as compared to control *hPXR* mice, and in *mPxr*^−/−^ mice treated with rifampicin and isoniazid. Differential protein expression was performed using a moderated T-test implemented in linear models for microarray (LIMMA). LIMMA was used to perform differential gene expression analysis. A cutoff *p*-value of 0.05 was used as the differential cutoff. Fishers-exact test using the up-regulated and down-regulated genes were tested against GO-Biological Process (BP) (**B**) and GO-Molecular Function (MF) (**C**) gene sets. An enrichment score was calculated based on the -log20(p-value). Log20 was chosen to normalize 0.05 to a score of 1.0 as a reference. Red indicates up-regulated proteins and blue indicates down-regulated proteins. hPXR_IvsCNTRL = Isoniazid-treated *hPXR* mice compared to control *hPXR* mice; hPXR_RIF.INHvsCNTRL = Rifampicin and isoniazid-treated *hPXR* mice compared to control *hPXR* mice; hPXR_RvsCNTRL = Rifampicin-treated *hPXR* mice compared to control *hPXR* mice; KO_R_IvsCNTRL = Rifampicin and isoniazid-treated *mPxr*^−/−^ mice compared to rifampicin and isoniazid treated *hPXR* mice.

We used 8-to-12-week-old *hPXR* transgenic and *mPxr*^−/−^ C57BL/6 mice for all in vivo experiments [28]. The mice were provided with food (Purina; St. Louis, MO, USA) containing rifampicin (300 mg/kg in chow) and autoclaved water containing isoniazid (1.2 g/L in water) ad libitum for 6 months (Figure 1A). We collected sera weekly to monitor liver function. All animal studies were approved by the Institutional Animal Care and Use Committee at St. Jude Children’s Research Hospital (St. Jude), and mice were housed in Association for Assessment and Accreditation of Laboratory Animal Care-accredited facilities. Quantification of liver function tests from plasma and sera was performed on the ABX Pentra instrument from Medline Industries (Northfield, IL, USA). HORIBA Enzyme Assays from Fisher Scientific (Waltham, MA, USA) were used to analyze alkaline phosphatase (ALP) (Catalogue # 23600417), alanine aminotransferate (ALT) (Catalogue # 23600418), direct bilirubin (Catalogue # 23600426), and total bilirubin (Catalogue # 23600430). Liver damage was evaluated by hematoxylin and eosin staining, and pathologic scoring was performed by board-certified pathologists.

### 2.4. Immunoblot Analysis

Livers were collected, flash frozen, and lysed [50 mM 4-(2-hydroxyethyl)-1-piperazineethanesulfonic acid (HEPES), pH 8.5, 8 M urea, and 0.5% sodium deoxycholate], and 100 µg of protein was digested with LysC from Wako Chemicals (Richmond, VA, USA) in the presence of 1,4-dithiothreitol at an enzyme-to-substrate ratio of 1:100 for 2 h as described previously [32]. Then, the samples were diluted to a final concentration of 2 M urea with 50 mM HEPES, pH 8.5, and stored at −80°C until immunoblot analysis was performed. NuPAGE LDS loading buffer and reducing agent from Invitrogen (Carlsbad, CA, USA) were used to prepare protein lysates for electrophoresis. Protein were separated with NuPAGE 4%–12% Bis-Tris protein gels (Invitrogen, Carlsbad, CA, USA), and protein sizes were estimated by using the Kaleidoscope protein marker from Bio-Rad (Hercules, CA, USA). Proteins were transferred from gels to iBlot nitrocellulose membranes (Invitrogen) by dry transfer. Membranes were incubated for 1 h at room temperature (RT) in tris buffered saline with tween (TBST) blocking buffer (LI-COR, Lincoln, NE, USA) before probing with primary antibodies. Membranes were incubated with primary antibodies overnight at 4°C and then incubated with secondary antibodies for 1 h at RT before visualization. Then, we stripped the membranes with NewBlot Nitro stripping buffer (LI-COR), incubated them with an anti–β-actin primary antibody (a5441; Sigma-Aldrich, St. Louis, MO, USA; diluted 1:1000) overnight at 4°C, and then incubated them with a secondary antibody for 1 h at RT. We visualized the protein bands with an Odyssey infrared imager (LI-COR Biosciences) and determined the relative intensity of each band by normalizing their intensities to that of the actin band [27,33].

### 2.5. Proteomic Profiling

Mouse livers were prepared as described for immunoblotting, and the profiling was performed following the optimized procedure [34]. To generate peptides for liquid chromatography (LC) and mass spectrometry (MS) analyses, the protein samples were digested with trypsin from Promega (Madison, WI, USA) after resuspension in 50 mM HEPES at an enzyme-to-substrate ratio of 1:50 for 3 h. The resulting peptides were reduced by adding 1 mM 1,4 –dithiothreitol for 30 min at RT and were alkylated with 10 mM iodoacetamide for 30 min at RT in the dark. The reaction was quenched by adding trifluoroacetic acid. This acidified peptide mixture was desalted by C18 cartridges from Harvard Apparatus (Holliston, MA, USA). The desalted eluates were dried and resuspended in 50 mM HEPES, pH 8.5.

Samples were labeled with 11-plex TMTs from Thermo Fisher (Waltham, MA, USA), according to the manufacturer recommendations. After labeling, the samples were combined, desalted, and fractionated with an off-line basic pH reverse phase C18 LC using high-performance liquid chromatography (HPLC; Agilent 1220) from Agilent Technologies (Santa Clara, CA, USA). The collected concatenated fractions were dried, resuspended in 5% formic acid, and analyzed by acidic pH reverse-phase LC-MS/MS. The samples were fractionated with a nanoscale capillary reverse-phase C18 column on a nanoAcquity HPLC from the Waters Corporation (Millford, MA, USA). The eluents were ionized by electrospray ionization and detected with an inline Orbitrap Fusion MS instrument from Thermo Fisher (Richmond, VA, USA). MS was performed in a data-dependent mode, with a survey scan (60,000 resolution, 1 × 10^6^ automatic gain control target and 50-microsecond maximal ion time) and 20 MS/MS high resolution scans (60,000 resolution, 1 × 10^5^ automatic gain control target and 150 microsecond maximal ion time, 38 high-energy collision-induced dissociation normalized energy, 1 m/z isolation window, and 20-s dynamic exclusion).

Raw mass spectra were processed by the JUMP program [35]. The resultant data were compared to the UniProt mouse database and concatenated with a reversed protein sequence decoy database. Searches were performed with a mass tolerance of 25 ppm for precursor ions and 15 ppm mass tolerance for fragment ions, fully tryptic restriction with two maximum missed cleavages, three maximum modification sites, and assignment of the *a*, *b,* and *y* ions. TMT tags on lysine residues and N-termini (+229.162932 Da) and the carbamidomethylation of cysteine residues (+57.021 Da) were used for determining static modifications, and methionine oxidation (+15.99492 Da) was considered a dynamic modification. Mass spectra were filtered by mass accuracy and matching scores to reduce the false discovery rate (FDR) to approximately 1%. The proteins were quantified by summarizing reporter ion counts across all matched peptide spectrums with the JUMP software suite [36]. Clustering analysis was performed with R v.3.0.1.

### 2.6. Statistical Analysis

We pooled data from three independent experiments and performed statistical analyses with GraphPad Prism 7.0 software. Venn diagrams were generated by using the Venny software v.2.0.2. All parametric data were expressed as the mean and standard error. To compare group means for qRT-PCR analysis, we used a two-way analysis of variance (ANOVA) and Tukey post hoc analysis. For immunoblot analysis, we used a one-way ANOVA and Dunnett post hoc analysis. We determined the changed proteins using the following steps based on previous published cutoffs with slight modifications [37]. (1) We applied a commonly used cutoff of *p* value (0.05) based on one-way ANOVA. (2) We used an additional cutoff the magnitude of change (*z* score > 2). The *z* score was defined by evaluating standard deviation of the experiments through analyzing biological replicates (null experiments) and then applying the standard deviation for *z* score conversion [36]. The *z* score of 2 was usually equivalent to > 1.15-relative change for up-regulated proteins and < 0.85-relative change for down-regulated proteins unless otherwise noted. (3) We performed permutation analysis (n = 1000 permutations) and found that the FDR was below 20%. The mass spectrometry proteomics data have been deposited to the ProteomeXchange Consortium via the PRIDE [38] partner repository with the dataset identifier PXD019505.

## 3. Results

### 3.1. Hepatopathic Changes

**Figure 2:**
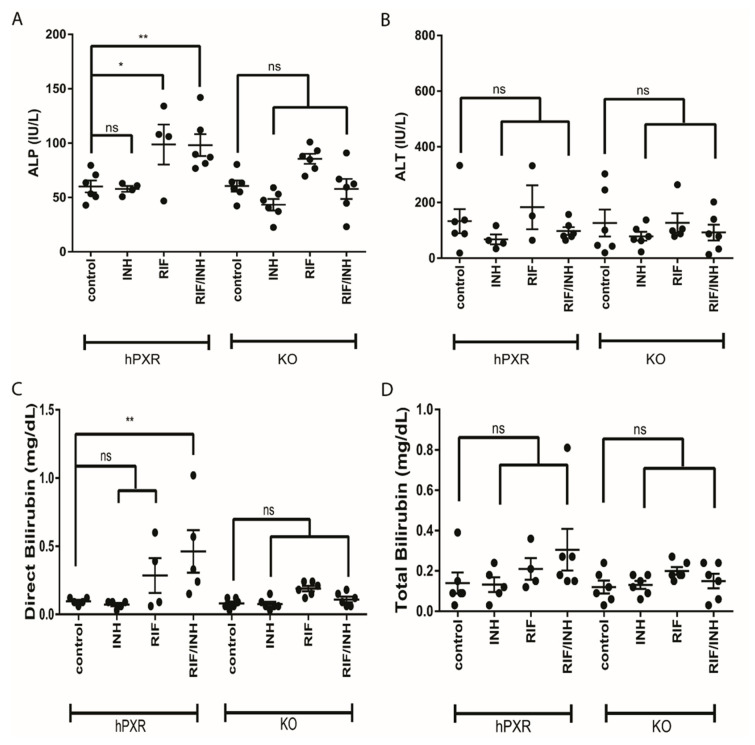
Liver function tests from human pregnane X receptor (*hPXR*) and *mPxr*^−/−^ knockout (KO) mice treated with rifampicin (RIF) and isoniazid (INH). Sera were analyzed for markers of hepatotoxicity before liver collection. (**A**) Alkaline phosphatase (ALP), (**B**) alanine aminotransferate (ALT), (**C**) direct bilirubin, and (**D**) total bilirubin are shown. Data are expressed as the mean ± SEM. One-way ANOVA followed by Dunnett post hoc analysis was used to compare group means. ** *p* < 0.01, * *p* < 0.05, and ns (not significant).

**Figure 3:**
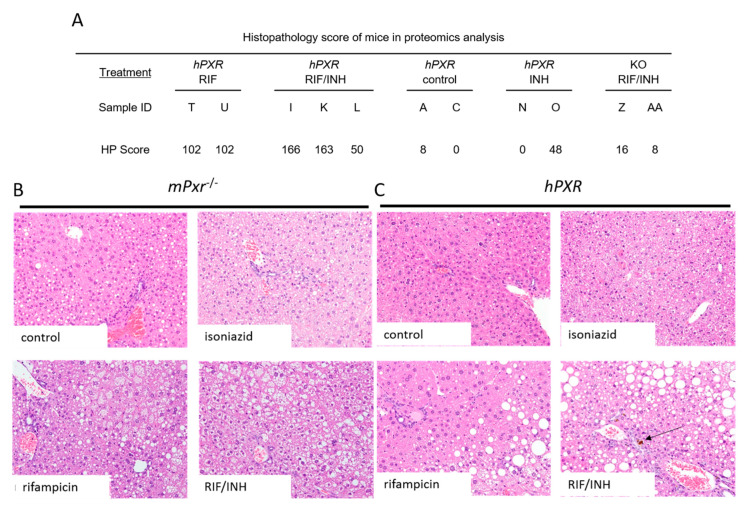
Histopathology of mouse livers. Semiquantitative histopathology scoring was performed by a board-certified veterinary pathologist. (**A**) Total hepatopathology scores (HP) were determined by scoring inflammation, karyomegaly, steatosis, bile pigment, and necrosis. Hematoxylin and eosin staining of *hPXR* (**B**) and *mPxr*^−/−^ (**C**) mouse livers (20×), arrows indicate bile plugs.

We observed hepatotoxic changes in *mPxr*^−/−^ and *hPXR* transgenic C57BL/6 mice treated for 6 months with combinations of 300 mg/kg rifampicin in chow and 1.2 g/L isoniazid in water (Figure 1A). This was evidenced by markedly increased direct bilirubin (Figure 2C) and alkaline phosphatase (ALP; Figure 2A) in *hPXR* mice treated with both rifampicin and isoniazid. We identified protein changes specific to mouse strain and drug treatment (Supplemental Figure S1). Rifampicin increased CYP3A protein levels in an *hPXR*-dependent manner (Supplementary Figure S2, S3, and S4C), and isoniazid decreased FECH protein levels (Supplementary Figures S2, S3, and S4A) and increased ALAS1 (Supplementary Figures S2, S3, and S4B). Histologic examination revealed the presence of bile plugs (Figure 3C) and higher hepathopathologic (HP) scores (Figure 3A) in *hPXR* mice treated with both rifampicin and isoniazid. These changes are consistent with previous reports of *hPXR*-dependent hepatotoxicity in mice treated with isoniazid and rifampicin [27,39,40].

### 3.2. Quantitative Analysis of the Liver Proteome

We identified and quantified 8466 proteins in the livers of *mPxr^–^*^/–^ and *hPXR* mice (Figure 1A). Clustering analysis revealed similar protein changes within groups (Figure 1B,C, and Supplementary Figures S5 and S6). The protein changes observed in *hPXR* mice treated with the drug combination and not in the *mPxr^−/−^* mice are most likely *hPXR*-specific. The proteins that were changed in both strains most likely represent *hPXR*-independent effects. Supplementary Figure S1 summarizes these protein changes in the different mouse strains (*hPXR* or *mPxr–/–* mice) and drug treatment groups (also see Supplementary Figure S6; Supplementary Tables S12, S13, and S14). Therefore, distinct protein changes occurred between the treatment groups, which we further analyzed to identify the proteins that mediate hPXR-induced isoniazid and rifampicin toxicity.

#### 3.2.1. Heme Biosynthesis and Degradation

Heme biosynthesis disruption is reported to occur after rifampicin and isoniazid treatment in mice and is associated with increased liver levels of PPIX [40]. ALAS1 increased at a 1.9-relative change in *hPXR* mice treated with both rifampicin and isoniazid, a 2.6-relative change in *hPXR* mice treated with isoniazid alone, and a 1.4-relative change in *mPxr*^−/−^ mice treated with the rifampicin and isoniazid combination. However, these changes did not significantly differ across the groups (Supplementary Table S1; *p* = 0.28). FECH decreased (*p* < 0.0001) in all groups, with a greater decrease (0.39-relative change to 0.5-relative change) in mice treated with isoniazid alone or in combination with rifampicin.

We found other heme biosynthesis proteins that changed with antitubercular therapy (Supplementary Table S1). These changes can be associated with an accumulation of toxic compounds related to heme biosynthesis, such as PPIX and others. Uroporphyrinogen-III synthase (UROS) significantly (*p* = 0.012) increased at a 1.19-relative change in *hPXR* mice treated with both rifampicin and isoniazid and a 1.31-relative change in *hPXR* mice treated with rifampicin. UROS also increased at a 1.38-relative change in *mPxr*^−/−^ mice treated with both rifampicin and isoniazid. These data suggest that UROS is increased by rifampicin in an *hPXR*-independent manner. Protoporphyrinogen oxidase (PPOX) significantly decreased (*p* = 0.013) with all treatments in an *hPXR*-independent manner by 0.69- to 0.85-relative change, and this effect was more pronounced in *hPXR* mice treated with both rifampicin and isoniazid (0.69-relative change) than in *mPxr*^−/−^ mice (0.83-relative change). Biliverdin reductase (BLVRA) significantly decreased (0.82- to 0.92-relative change) in all treatment groups, independent of *hPXR* status (*p* = 0.032). These data are consistent with previous reports of ALAS1 up-regulation and FECH down-regulation due to isoniazid and its metabolites and demonstrate a potential for the accumulation of toxic heme metabolites [27,39,40].

Ferritin light chain (FTL1) significantly decreased (*p* = 8.42 × 10^−5^) in all treatment groups (Supplementary Table S3). FTL1 was most severely decreased in *hPXR* mice treated with both rifampicin and isoniazid (0.28-relative change), followed by *hPXR* mice treated with isoniazid alone (0.36-relative change). ABCB7 significantly decreased (0.83-relative change) in *hPXR* mice treated with rifampicin and isoniazid (*p* = 0.019) (Supplementary Table S3). ABCB7 also decreased in *hPXR* mice treated with rifampicin (0.79-relative change) and modestly decreased in *mPxr*^−/−^ mice treated with both rifampicin and isoniazid (0.91-relative change). These data are consistent with reports of anemia in patients with tuberculosis who are treated with antitubercular drugs [7,8,10].

#### 3.2.2. CYP Induction

CYP2B10 is the most up-regulated CYP and significantly increased (*p* = 0.0003) with rifampicin treatment alone (2.49-relative change) and with the rifampicin and isoniazid combination treatment (4.95-relative change) in *hPXR* mice (Supplementary Table S4). CYP3A11—the mouse CYP3A4 ortholog [41]—significantly increased (*p* = 0.0008) in *hPXR* mice treated with rifampicin (4.64-relative change) and treated with both rifampicin and isoniazid (3.89-relative change). Several CYPs decreased with isoniazid treatment: CYP1A2 (0.48-relative change), CYP2A12 (0.78-relative change), CYP2A5 (0.62-relative change), and CYP2D26 (0.71-relative change). CYP2E1 significantly increased (*p* = 0.00035) in *hPXR* mice treated with the rifampicin and isoniazid combination (1.22-relative change) or isoniazid alone (1.18-relative change). CYP2E1 also increased in *mPxr*^−/−^ mice treated with both rifampicin and isoniazid (1.47-relative change). Polymorphisms associated with increased CYP2E1 activity in humans increase the incidence of drug-induced liver injury (DILI) [42,43,44]. Inhibiting CYP2E1 is protective against isoniazid-induced hepatotoxicity in mice [45]. CYP2E1 wild-type mice are more susceptible to DILI than are mice lacking the gene [46]. Therefore, CYP2E1 upregulation by rifampicin in an *hPXR*-independent manner may contribute to DILI.

The nuclear receptors HNF1A, NR3C, and NR5A significantly decreased in *hPXR* mice (Supplementary Table S2), but other nuclear receptors were not significantly changed, suggesting that the changes observed in target genes (i.e., CYP enzymes) were caused by the activation of these nuclear receptors rather than a change in the expression of the receptors.

#### 3.2.3. Oxidative Stress, Wound Healing, and Inflammation

Many isoforms of glutathione S transferase mu (GSTM), which detoxify electrophilic compounds and cluster on chromosome 1p13.3, significantly changed in *hPXR* mice (Supplementary Table S5; *p* < 0.0001). GSTM-1, -2, -3, -4, -5, -6, and -7 increased relative to the change in *hPXR* (1.59- to 3-relative change) and *mPxr*^−/−^ (1.16- to 1.61-relative change) mice treated with the rifampicin and isoniazid combination, decreased (0.79- to 0.87-relative change) in *hPXR* mice treated with isoniazid alone, and increased with the highest magnitude in *hPXR* mice treated with rifampicin alone (1.55-relative change to 4.5-relative change). GSTM-1 is protective in humans treated with antitubercular drugs, and null alleles are associated with a higher incidence of hepatotoxicity [2,47,48]. Catalase (CAT) also significantly increased (*p* = 0.038) in *hPXR* mice treated with both rifampicin and isoniazid (1.16-relative change) or rifampicin alone (1.45-relative change). NAD(P)H dehydrogenase quinone 1 (NQO1) significantly increased (*p* < 0.001) in *hPXR* mice treated with the rifampicin and isoniazid combination (1.52-relative change) or rifampicin alone (2.6-relative change). In *mPxr*^−/−^ mice treated with both rifampicin and isoniazid, NQO1 increased at a 1.92-relative change. In contrast, NQO1 decreased in *hPXR* mice treated with isoniazid alone (0.87-relative change). The decreased expression of GSTM isoforms may potentiate the hepatotoxicity of antitubercular therapy, whereas the increase in catalase and NQO1 may be protective [49].

Several proteins involved in wound healing and inflammation were affected by antitubercular treatment in our analysis. Protein–glutamine gamma-glutamyltransferase 2 (TGM2) significantly increased (Supplementary Table S6; *p* = 0.0058) in *hPXR* mice treated with both rifampicin and isoniazid (1.12-relative change) or rifampicin alone (1.19-relative change). In contrast, TGM2 was down-regulated in *hPXR* mice treated with isoniazid alone (0.81-relative change) and in *mPxr*^−/−^ mice treated with the rifampicin and isoniazid combination (0.88-relative change). Collagen alpha-1(IV) (COL4A1) and collagen alpha-1 (VI) (COL6A1) significantly increased (*p* < 0.01) in *hPXR* mice treated with both rifampicin and isoniazid (1.27- and 1.10-relative change, respectively). Annexin A5 (ANXA5) significantly increased (*p* = 0.032) at a 1.56-relative change in *hPXR* mice treated either with the rifampicin and isoniazid combination or rifampicin alone. ANXA5 also increased in *mPxr*^−/−^ mice treated with both rifampicin and isoniazid (1.22-relative change). The up-regulation of these proteins suggests that tissue injury was induced by the antitubercular drugs [50,51,52].

#### 3.2.4. [Fe–S] Cluster-Containing Proteins

Many [2Fe–2S] cluster-containing proteins are decreased by isoniazid, but we did not observe a significant global down-regulation of [Fe–S] cluster-containing proteins in response to isoniazid (Supplementary Table S7). NADH dehydrogenase ubiquinone iron–sulfur protein 8 (NDUFS8) significantly decreased (*p* = 0.0012) in all treatment groups, but with a lower magnitude of decrease in mice treated with isoniazid alone. Aldehyde oxidase 1 (AOX1) significantly decreased (*p* = 0.0023) with isoniazid alone (0.82-relative change), and xanthine dehydrogenase (XDH) significantly decreased (*p* = 0.0079) at a 0.70-relative change in all *hPXR* groups. The succinate dehydrogenase ubiquinone iron–sulfur subunit (SDHB) significantly decreased (*p* = 0.036) in *hPXR* mice treated with the rifampicin and isoniazid combination (0.86-relative change), but to a lesser extent in the other groups. These changes in SDHB may be *hPXR*-mediated by rifampicin and further decreased by isoniazid administration. NADH dehydrogenase ubiquinone iron–sulfur protein 7 (NDUFS7) significantly increased (*p* = 0.012) in *hPXR* mice treated with isoniazid alone (1.14-relative change) and in *mPxr*^−/−^ mice treated with both rifampicin and isoniazid (1.20-relative change). NADH dehydrogenase ubiquinone iron–sulfur protein 4 (NDUFS4) significantly increased (*p* = 0.033) in all groups except in *hPXR* mice treated with the rifampicin and isoniazid combination. Iron-responsive element-binding protein 2 (IREB2) significantly increased (*p* = 0.039) in all treatment groups at a 1.18- to 1.42-relative change. Ferrodoxin 1 (FDX1) (Supplementary Table S8) and FECH (Supplementary Table S1) significantly decreased with isoniazid treatment.

Isoniazid may affect a subset of [Fe–S] cluster-containing proteins rather than globally affecting these proteins. The NADH dehydrogenase ubiquinone, also termed complex 1, is composed of multiple subunits and contains eight [Fe–S] clusters: multiples of [2Fe–2S], [4Fe–4S], and [3Fe–4S]. FDX contains a [2Fe–2S] cluster [53]. SDHB contains three [Fe-S] clusters—[2Fe–2S], [4Fe–4S], and [3Fe–4S]—and was down-regulated by rifampicin and isoniazid combination treatment in *hPXR* mice in our analysis [54]. AOX1 and XDH (Supplementary Table S7) are [2Fe–2S]-containing proteins that were also down-regulated by isoniazid treatment in our analysis [55,56]. FECH contains a [2Fe–2S] cluster [57]. NDUFS8 was decreased and contains two [4Fe–4S] clusters, but the decrease was not mediated by isoniazid [58]. The effect of isoniazid on [Fe–S] clusters may be specific to [2Fe–2S] clusters.

#### 3.2.5. [Fe–S] Cluster Assembly Machinery

The effects of isoniazid on heme biosynthesis, iron metabolism, and [Fe–S] cluster-containing proteins may also be associated with an effect on [Fe–S] cluster assembly machinery. Cysteine desulfurase (NFS1) significantly decreased (*p* < 0.05) in *hPXR* mice treated with the rifampicin and isoniazid combination (0.89-relative change) or rifampicin alone (0.84-relative change). NFS1 also decreased to a lesser degree in *mPxr*^−/−^ mice treated with both rifampicin and isoniazid (0.90 relative change). However, NFS1 changed less than 4% in *hPXR* mice treated with isoniazid (Supplementary Table S8). FDX1 significantly decreased (*p* = 0.0016) in all treatment groups and decreased to a greater degree with isoniazid alone (0.77-relative change) than with rifampicin alone (0.84-relative change). However, FDX1 decreased the most with the combination of rifampicin and isoniazid in *hPXR* mice (0.64-relative change). FDX1 also decreased in *mPxr*^−/−^ mice treated with both rifampicin and isoniazid (0.79-relative change). The iron–sulfur cluster assembly 1 homolog (ISCA1) significantly decreased (*p* = 0.0037) from 0.66- to a 0.81-relative change in all treatment groups. BolA-like protein 3 (BOLA3) significantly decreased (*p* = 0.012) in *hPXR* mice treated with isoniazid (0.91-relative change) or the combination of rifampicin and isoniazid (0.89-relative change). However, BOLA3 increased in *hPXR* mice treated with rifampicin alone (1.12-relative change). The greatest effect of antitubercular therapy on [Fe–S] cluster assembly machinery in our analysis was on FDX1, which reduces CYPs and serves as an iron donor in the early steps of [Fe–S] cluster assembly [53].

#### 3.2.6. Vitamin B_6_ Metabolism

Isoniazid treatment is associated with vitamin B_6_-dependent peripheral neuropathy, pellagra, and sideroblastic anemia [8,14,15,20]. A recent study demonstrated that isoniazid and vitamin B_6_ conjugate to form pyridoxal isonicotinoyl hydrazone (PIH) [27]. If enough vitamin B_6_ is depleted by conjugation with isoniazid, clinical vitamin B_6_ deficiency may occur. This study also found that pyridoxal 5′-phosphate formed more PIH when combined with isoniazid than did nonphosphorylated forms of vitamin B_6_ [27]. The vitamin B_6_ metabolism enzymes pyridoxal kinase (PDXK) and pyridoxal-dependent decarboxylase domain-containing protein 1 (PDXDC1) did not significantly change in *hPXR* mice (Supplementary Table S9). Pyridoxal phosphate phosphatase (PDXP) significantly increased (*p* = 0.00049) at a 1.49-relative change in *hPXR* mice treated with both rifampicin and isoniazid, a 2.18-relative change in *hPXR* mice treated with rifampicin alone, and a 1.34-relative change in *mPxr*^−/−^ mice treated with the rifampicin and isoniazid combination. PDXP significantly increased with rifampicin treatment alone and with the rifampicin and isoniazid combination in *hPXR* mice and in *mPxr*^−/−^ mice (Supplementary Table S9). PDXP dephosphorylates pyridoxal 5′ phosphate to 4-pyridoxic acid but also dephosphorylates pyridoxine 5′ phosphate and pyridoxamine 5′ phosphate [59]. An increased dephosphorylation of vitamin B_6_ by upregulated PDXP in response to rifampicin may increase the amount of PIH formed and subsequently decrease vitamin B_6_ levels.

#### 3.2.7. Homocysteine Metabolism

Antitubercular therapy is associated with homocysteinemia [12,60,61]. Proteins involved in homocysteine metabolism were affected by antitubercular therapy in our analysis (Supplementary Table S10). Methionine synthase significantly increased (*p* < 0.05) at a 1.26-relative change in *hPXR* mice treated with rifampicin. Betaine homocysteine S-methyltransferase 1 significantly increased (*p* < 0.05) in all treatment groups. Cystathionine β-synthase (CBS) significantly decreased (*p* < 0.01) in all treatment groups, but *hPXR* mice treated with both rifampicin and isoniazid or rifampicin alone (0.62-relative change) exhibited the largest decrease. Cystathionine γ-lyase significantly decreased (*p* < 0.01) at a 0.87-relative change in *hPXR* mice treated with rifampicin alone. Lower levels of CBS may result from increased serum homocysteine [62,63].

#### 3.2.8. Tryptophan Metabolism

Proteins involved in tryptophan and vitamin B_3_ metabolism were affected by antitubercular therapy in our analysis (Supplementary Table S11). The disruption of tryptophan metabolism can result in niacin deficiency. The 3-hydroxyanthranilate 3,4-dioxygenase (HAAO) significantly decreased (*p* < 0.01) in *hPXR* mice treated with rifampicin alone (0.91-relative change) or with the combination of rifampicin and isoniazid (0.91-relative change) but increased in *mPxr*^−/−^ mice (1.14-relative change). The kynurenine/alpha-aminoadipate aminotransferase (AADAT) significantly decreased (*p* < 0.01) in all treatment groups, with the greatest decrease occurring in *hPXR* mice treated with rifampicin alone (0.56-relative change). Kynurenine 3-monooxygenase (KMO) significantly decreased (*p* < 0.05) in *hPXR* mice treated with rifampicin alone (0.78-relative change) or with the combination of rifampicin and isoniazid (0.82-relative change), although isoniazid modestly decreased (0.91-relative change) KMO. In contrast, KMO increased (a 1.11-relative change) in *mPxr*^−/−^ mice. Nicotinate phosphoribosyltransferase (NAPRT) significantly decreased (*p* < 0.01) in *hPXR* mice treated with rifampicin alone (0.83-relative change) or the combination of rifampicin and isoniazid (0.87-relative change) and in *mPxr*^−/−^ mice treated with both rifampicin and isoniazid (0.79-relative change). The HAAO, AADAT, KMO, and NAPRT all decreased with rifampicin treatment alone. Specifically, HAAO and KMO decreased with rifampicin treatment in *hPXR* mice but not in *mPxr*^−/−^ mice. Therefore, the effect of rifampicin on these proteins may be *hPXR* dependent. AADAT and KMO decreased with isoniazid treatment, but the magnitude of the decrease was higher with rifampicin treatment. These changes may result in systemic niacin depletion.

## 4. Discussion

We observed markedly elevated ALP and direct bilirubin levels, with histopathologic findings of bile plugs, in hPXR mice. These findings suggest a cholestatic pattern of DILI in response to rifampicin and isoniazid treatment, which recapitulates a previously reported milder phenotype of *hPXR*-dependent rifampicin- and isoniazid-induced DILI in mice [39,64,65,66]. Analysis of the liver proteome during the early stages of DILI may diminish the confounding effects of advanced hepatopathology in the later stages of DILI on the liver proteome. The control *hPXR* mice we used in our proteomic analysis had lower levels of hepatotoxicity than did *hPXR* mice treated with rifampicin and isoniazid, which had higher histopathologic scores. Correspondingly, the wound-healing proteins ANXA5, COL4A1, and TGM2 were increased in *hPXR* mice treated with both rifampicin and isoniazid. Therefore, our findings highlight the toxicoproteomic changes seen in *hPXR*-mediated antitubercular DILI without obfuscation by extensive tissue death.

Our profiling analysis revealed many changes in the proteins involved in relieving oxidative stress. Multiple GSTM isoforms were increased by rifampicin in an *hPXR*-dependent manner. GSTM-1 is reported to be protective in humans treated with rifampicin and isoniazid. Null alleles of *GSTM1* in humans may lead to increased DILI incidence [47,48,67,68,69,70,71,72]. In our study, rifampicin increased catalase in an *hPXR*-dependent manner, which may be protective by neutralizing H_2_O_2_. NQO1 is protective against acetaminophen (APAP)-induced hepatotoxicity and is increased in APAP-associated DILI [49] and primary biliary cirrhosis [73]. We found that NQO1 was increased in an *hPXR*-independent manner due to rifampicin and isoniazid combination treatment and to a greater extent from rifampicin treatment alone. Therefore, the rifampicin induction of *hPXR* may have imparted a protective effect on these oxidative stress proteins.

Cholestatic liver injury caused by rifampicin and isoniazid treatment was associated with PPIX accumulation and heme biosynthesis disruption, upregulated ALAS1, and downregulated FECH [27,39,40]. Furthermore, the effects on the heme biosynthetic pathway were due to the isoniazid metabolites hydrazine and PIH [27]. PIH is an iron chelator that forms from an enzyme-independent reaction between multiple vitamin B_6_ orthologs and isoniazid. It also decreases FECH protein levels in an iron-dependent manner. Likewise, hydrazine increases ALAS1 at the mRNA and protein levels [27]. We and others have demonstrated ALAS1 up-regulation and FECH down-regulation in isoniazid-treated mice. Additional changes in the heme biosynthetic proteins BLVRA, PPOX, and UROS may also play a role in PPIX accumulation in an *hPXR*-independent manner. Rifampicin and isoniazid may induce PPIX formation to cause hepatotoxicity and exacerbated acute intermittent porphyria [5,6].

Isoniazid causes peripheral neuropathy secondary to vitamin B_6_ depletion [14,16], with the conjugation of isoniazid to vitamin B_6_ species (pyridoxal, pyridoxine, and pyridoxal 5′ phosphate) resulting in decreased vitamin B_6_ levels in humans and rats and the formation of PIH [8,15,21,22,27]. Vitamin B_6_ deficiency can lead to confusion, mood changes, seizures, stomatitis, cheilosis, and seborrheic dermatitis [74,75]. Rifampicin-mediated increased PDXP may lead to increased nonphosphorylated species of vitamin B_6_, which form more PIH than phosphorylated species do [27,59]. We found this increase was most pronounced in *hPXR* mice treated with rifampicin alone. An increase of isoniazid-reactive forms of vitamin B_6_ may contribute to the depletion of systemic vitamin B_6_ stores. Therefore, rifampicin may disrupt vitamin B_6_ metabolism in an hPXR-dependent manner, and isoniazid has been shown to disrupt vitamin B_6_ metabolism in an hPXR-independent manner, previously [27].

Patients with tuberculosis are at an increased risk of deep vein thrombosis (DVT), pulmonary embolism (PE), and ischemic stroke [76,77,78], and antitubercular therapy may increase these risks, in addition to increased myocardial infarction risk [11,12,13,60]. Additionally, antitubercular therapy may cause homocysteinemia, which increases coagulability [12,60,61]. We found decreased cystathionine β-synthase in all treatment groups in an *hPXR*-independent manner. Cystathionine β-synthase is vitamin B_6_-dependent and catalyzes the conversion of homocysteine to cystathionine. The decreased activity of this enzyme increases homocysteine levels [62,63]. We also found decreased cystathionine γ-lyase, which catalyzes the vitamin B_6_-dependent conversion of cystathionine to cysteine, in *hPXR* mice treated with isoniazid in an *hPXR*-dependent manner. Decreases in these proteins may result in increased serum levels of homocysteine, which contribute to coagulopathy.

FTL1 was decreased in all treatment groups in an *hPXR*-independent manner, but the highest magnitude of decrease was in *hPXR* mice treated with both rifampicin and isoniazid. Low ferritin levels are a clinical sign of low systemic iron levels and may be caused by long-term exposure to an iron chelator. Deficiency of the iron transporter ABCB7 is associated with sideroblastic anemia, with ring sideroblasts from intracellular iron accumulation in erythrocytes [79]. ABCB7 exports [2Fe–2S] clusters out of the mitochondria and into the cytosol, and ABCB7 deficiency causes an iron-deficient phenotype and mitochondrial iron accumulation [79,80]. Indeed, sideroblastic anemia responsive to vitamin B_6_ administration is reported with antitubercular therapy [8,9,10], and multiple types of anemia (including iron-deficiency anemia) occur with antitubercular therapy [81,82,83]. These data and previous reports suggest that isoniazid and rifampicin lead to an iron-deficient state, with concomitant changes in heme biosynthesis regulation.

A labile iron–sulfur [2Fe–2S] cluster is present in animal FECH but is absent in the analogous plant, bacterial, and yeast enzymes [84,85,86]. However, in mammals, when this structure is targeted by nitric oxide synthases, the degradation of FECH is increased [87,88,89]. The [2Fe–2S] cluster in FECH is most likely required for recycling rather than catalysis. Iron-limiting conditions decrease FECH protein levels by forming an unstable protein with a half-life of approximately 1 h, as compared with a half-life over 35 h in iron-replete conditions [90]. Therefore, an iron-limiting condition in the presence of PIH may lead to the formation of a FECH protein that is less stable than the protein formed in iron-replete conditions.

Iron-limiting conditions may also be associated with the down-regulation of other [Fe–S] cluster-containing proteins or [Fe–S] cluster assembly machinery proteins. The PIH-mediated FECH down-regulation of FECH is iron dependent [27]. The iron-limiting condition induced by deferoxamine reduces the half-life of FECH formed after treatment [90]. Indeed, we found two [Fe–S]-containing proteins down-regulated by isoniazid that contain multiple [Fe–S] clusters, some of which are [2Fe–2S] clusters. Multiple proteins containing [2Fe–2S] clusters were down-regulated by the rifampicin and isoniazid combination treatment independent of *hPXR*. This may be due to a reduced availability of iron to form [2Fe–2S] clusters or downstream effects during iron–sulfur cluster assembly. BOLA3, FDX1, ISCA1, and NFS1 were decreased by the rifampicin and isoniazid combination treatment in both *hPXR* and *mPxr*^−/−^ mice. Whether these changes are direct effects of the drug treatment or are compensatory to other changes is unclear, but they do not appear to be caused by isoniazid treatment alone. The combination of rifampicin and isoniazid may decrease the ability to produce [Fe–S] clusters by affecting protein levels of cellular [Fe–S] cluster assembly machinery.

Hydrazine increases ALAS1 mRNA and protein [27] and is reported to N-alkylate, deactivate, and increase the turnover of CYP enzymes [91,92,93,94]. In addition to rifampicin-upregulated CYP enzymes, several CYP species were decreased by isoniazid in our analysis. Heme negatively regulates ALAS1 at the transcriptional, translational, and post-translational levels [95,96,97,98,99]. If hydrazine decreases the regulatory heme pool by the N-alkylation of cellular hematoproteins, this would result in ALAS1 up-regulation at both the protein and mRNA levels.

Rifampicin decreased the kynurenine pathway enzymes HAAO, AADAT, and KMO. The decreased HAAO and KMO may be mediated by *hPXR*. The kynurenine pathway catabolizes tryptophan to quinolinic acid for use in the nicotinamide adenine dinucleotide pathway. Deficiencies in this pathway disrupt tryptophan catabolism, and a nutritional deficiency in niacin or tryptophan causes vitamin B_3_ (niacin) deficiency [100,101]. Antitubercular therapy can interfere with tryptophan metabolism, leading to decreased vitamin B_3_ and pellagra, which is characterized by dermatitis, diarrhea, and dementia and can progress to death if untreated [18,19,102].

Our findings confirm and expand upon previous reports of antitubercular therapy disruption of the heme biosynthetic pathway [27,39,40]. Namely, antitubercular therapy affects proteins associated with [Fe–S] cluster assembly machinery, heme metabolism, homocysteine metabolism, iron metabolism, oxidative stress responses, tryptophan metabolism, vitamin B_3_ metabolism, and vitamin B_6_ metabolism. These findings may elucidate the pathophysiology of the antitubercular therapy complications of acute intermittent porphyria, anemia, DILI, drug–drug interactions, hypercoagulability (DVT, PE, ischemic strokes), pellagra, peripheral neuropathy, and porphyria cutanea tarda.

## 5. Conclusions

Isoniazid may play roles in such pathologic as hepatotoxicity, acute intermittent porphyria, anemia, pellagra (vitamin B_3_ deficiency), vitamin B_6_ deficiency, and hypercoagulable states (DVT, PE, or ischemic stroke). We observed hPXR-mediated changes in glutathione S-transferases, kynurenine pathway, vitamin B6 metabolism, and wound-healing proteins. We also observed hPXR-independent changes caused by rifampicin and isoniazid in [2Fe-2S] cluster-containing proteins, ABCB7, ferritin, and heme metabolism proteins. Our findings provide insight into the etiology of the mechanistic processes leading to these states after treatment with antitubercular therapy.

## Supplementary Materials

The following are available online at https://www.mdpi.com/2073-4409/9/7/1654/s1, Figure S1: Venn diagrams of protein expression changes, Figure S2: Protein expression of mouse *mPxr*–/– liver samples considered for proteomics analysis, Figure S3: Protein expression in mouse *hPXR* liver samples considered for proteomics analysis, Figure S4: Quantification of Western blots of mouse livers selected for proteomics analysis, Figure S5: Hierarchical clustering analysis for mouse liver proteomic profiling, Figure S6: Heatmap of selected proteins; Table S1: Relative change in proteins associated with heme biosynthesis and degradation, Table S2: Relative change in protein nuclear receptors, Table S3: Relative change in proteins associated with iron metabolism, Table S4: Relative change in CYPs and steroid metabolism enyzmes, Table S5: Relative change in proteins associated with oxidative stress responses, Table S6: Relative change in proteins associated with wound healing and inflammation, Table S7: Relative change in [Fe–S] cluster-containing proteins, Table S8: Relative change in [Fe–S] cluster assembly machinery proteins, Table S9: Relative change in proteins associated with vitamin B6 metabolism, Table S10: Relative change in proteins associated with homocysteine metabolism, Table S11: Relative change in proteins associated with tryptophan metabolism, Table S12: Protein changes by mouse strain, Table S13: Proteins decreased by treatment in *hPXR* mice, Table S14: Proteins increased by treatment in *hPXR* mice.
