# Supplementary material for: Toxicoproteomic Profiling of hPXR Transgenic Mice Treated with Rifampicin and Isoniazid
Source: Cells. 2020 Jul 9;9(7):1654. doi: 10.3390/cells9071654 (PMC7407182; doi:10.3390/cells9071654)
Supplement: Supplementary file 1 [file cells-09-01654-s001.pdf]

Supplementary Table S1. Relative change in proteins associated with heme biosynthesis and degradation.

		hPXR			***mPxr*$^{-/-}$**	
**Protein**	**Gene**	**RIF/INH**	**INH**	**RIF**	**RIF/INH**	***p* Value**
5-aminolevulinate synthase	*Alas1*	1.90	2.61	1.05	1.41	0.28
5-aminolevulinate synthase	*Alas2*	0.86	1.38	0.73	1.18	0.018
Delta-aminolevulinic acid dehydratase	*Alad*	0.96	1.00	1.02	0.95	0.75
Porphobilinogen deaminase	*Hmbs*	1.04	0.99	1.10	1.05	0.67
Uroporphyrinogen-III synthase	*Uros*	1.19	1.09	1.31	1.38	0.012
Uroporphyrinogen decarboxylase	*Urod*	0.92	1.03	0.94	0.92	0.33
Oxygen-dependent coproporphyrinogen-III oxidase,	*Cpox*	1.13	1.04	1.18	1.15	0.20
Protoporphyrinogen oxidase	*Ppox*	0.69	0.81	0.85	0.83	0.013
Ferrochelatase,	*Fech*	0.39	0.50	0.88	0.43	0.000002
Heme oxygenase 1	*Hmox1*	1.15	0.86	0.91	1.11	0.34
Heme oxygenase 2	*Hmox2*	0.96	0.98	0.89	0.88	0.22
Biliverdin reductase A	*Blvra*	0.84	0.92	0.82	0.92	0.032
UDP-glucuronosyltransferase 1-6	*Ugt1a6*	1.22	0.96	1.10	1.13	0.30
NADPH--cytochrome P450 reductase	*Por*	1.28	0.92	1.18	1.12	0.019

INH, isoniazid; RIF, rifampicin; RIF/INH, rifampicin and isoniazid.

Supplementary Table S2. Relative change in protein nuclear receptors.

Protein	Gene	*hPXR*			*mPxr*$^{-/-}$	
		**RIF/INH**	**INH**	**RIF**	**RIF/INH**	***p* Value**
Aryl hydrocarbon receptor	*Ahr*	1.09	0.91	1.00	1.26	0.092
Hepatocyte nuclear factor 1-alpha	*Hnf1a*	0.87	0.97	0.82	0.79	0.027
Hepatocyte nuclear factor 4-alpha	*Hnf4a*	0.95	1.05	0.97	1.08	0.20
Oxysterols receptor LXR-alpha	*Nr1h3*	0.94	1.16	1.03	1.02	0.42
Bile acid receptor	*Nr1h4*	1.05	1.17	0.98	1.19	0.12
Retinoic acid receptor RXR-alpha	*Rxra*	0.88	1.03	0.83	0.95	0.12
Peroxisome proliferator-activated receptor alpha	*Ppara*	0.85	0.96	0.79	0.94	0.64
Nuclear receptor subfamily 1 group D member 1	*Nr1d1*	0.32	0.32	0.51	0.90	0.37
Oxysterols receptor LXR-beta	*Nr1h2*	1.11	1.14	1.08	1.05	0.62
Bile acid receptor	*Nr1h4*	1.05	1.17	0.98	1.19	0.12
COUP transcription factor 1	*Nr2f1*	0.98	1.04	0.94	1.00	0.67
COUP transcription factor 2	*Nr2f2*	0.98	1.04	0.94	1.00	0.67
Nuclear receptor subfamily 2 group F member 6	*Nr2f6*	1.01	1.04	0.97	1.08	0.45
Glucocorticoid receptor	*Nr3c1*	0.91	1.03	0.88	0.96	0.049
Nuclear receptor subfamily 5 group A member 2	*Nr5a2*	0.80	1.00	0.83	0.97	0.016

INH, isoniazid; RIF, rifampicin; RIF/INH, rifampicin and isoniazid.

Supplementary Table S3. Relative change in proteins associated with iron metabolism.

		hPXR			***mPXr***$^{-/-}$	
**Protein**	**Gene**	**RIF/INH**	**INH**	**RIF**	**RIF/INH**	***p* Value**
Serotransferrin	*Tf*	1.33	1.04	1.15	1.11	0.144145
Transferrin receptor protein 1	*Tfrc*	1.28	0.98	1.13	1.14	0.46
Lactotransferrin	*Ltf*	0.40	0.38	0.38	0.27	0.036
Transferrin receptor protein 2	*Tfr2*	0.99	1.02	0.82	0.86	0.13
Hereditary hemochromatosis protein homolog	*Hfe*	0.93	0.92	0.90	0.94	0.70
Ferritin light chain 1	*Ftl1*	0.28	0.36	0.58	0.44	8.42E-05
Ferritin heavy chain	*Fth1*	0.47	0.63	0.70	0.59	0.097
Cytoplasmic aconitate hydratase	*Aco1*	1.05	1.00	1.08	1.00	0.53
ATP-binding cassette sub-family B member 7	*Abcb7*	0.83	0.98	0.79	0.91	0.019

INH, isoniazid; RIF, rifampicin; RIF/INH, rifampicin and isoniazid.

Supplementary Table S4. Relative change in CYPs and steroid metabolism enyzmes.

		hPXR			***mPxr***$^{-/-}$	
**Protein**	**Gene**	**RIF/INH**	**INH**	**RIF**	**RIF/INH**	***p* Value**
Cytochrome P450 1A2	*Cyp1a2*	0.60	0.48	1.47	0.53	0.037
Cytochrome P450 2A12	*Cyp2a12*	0.98	0.78	1.53	0.91	0.022
Cytochrome P450 2A5	*Cyp2a5*	1.77	0.62	2.93	1.01	0.00076
Cytochrome P450 2B10	*Cyp2b10*	4.96	0.91	2.49	0.98	0.00032
Cytochrome P450 2C29	*Cyp2c29*	1.17	1.02	1.56	0.67	0.038
Cytochrome P450 2D10	*Cyp2d10*	0.98	0.90	1.03	0.99	0.92
Cytochrome P450 2D26	*Cyp2d26*	0.76	0.71	1.13	0.75	0.0021
Cytochrome P450 2E1	*Cyp2e1*	1.22	1.18	0.93	1.47	0.00035
Cytochrome P450 2F2	*Cyp2f2*	0.61	0.61	0.75	0.52	0.028
Cytochrome P450 2J6	*Cyp2j6*	0.98	0.89	1.04	1.06	0.68
Cytochrome P450 3A11	*Cyp3a11*	3.89	1.18	4.64	1.31	0.00086
Cytochrome P450 3A25	*Cyp3a25*	1.64	0.95	1.36	1.15	0.037
Cytochrome P450 4A10	*Cyp4a10*	2.38	1.61	4.45	1.78	0.012
Cholesterol 7-alpha-monooxygenase	*Cyp7a1*	1.08	0.65	0.86	1.85	0.095
25-hydroxycholesterol 7-alpha-hydroxylase	*Cyp7b1*	0.49	0.55	0.39	0.64	0.12
Sterol 26-hydroxylase	*Cyp27a1*	0.70	0.73	0.82	0.80	0.032
Lanosterol 14-alpha demethylase	*Cyp51a1*	0.94	0.66	1.30	1.56	0.43
Hydroxymethylglutaryl-CoA synthase	*Hmgcs1*	1.51	1.29	1.74	2.53	0.036
Hydroxymethylglutaryl-CoA synthase	*Hmgcs2*	1.26	1.55	1.23	1.19	0.027
3-hydroxy-3-methylglutaryl-coenzyme A reductase	*Hmgcr*	1.56	1.27	1.51	1.74	0.35
3 beta-hydroxysteroid dehydrogenase type 4	*Hsd3b4*	0.20	1.07	0.10	0.48	0.0012
Corticosteroid 11-beta-dehydrogenase isozyme 1	*Hsd11b1*	0.99	1.00	0.83	1.09	0.75

INH, isoniazid; RIF, rifampicin; RIF/INH, rifampicin and isoniazid.

Supplementary Table S5. Relative change in proteins associated with oxidative stress responses.

Protein	Gene	*hPXR*			*mPxr*$^{-/-}$	
**Protein**	**Gene**	**RIF/INH**	**INH**	**RIF**	**RIF/INH**	***p* Value**
Glutathione synthetase	*Gss*	0.95	0.80	0.91	0.76	0.084
Phospholipid hydroperoxide glutathione peroxidase	*Gpx4*	1.44	1.05	1.56	1.23	0.0016
Glutathione S-transferase mu 1	*Gstm1*	1.59	0.80	2.50	1.19	7.23E-06
Glutathione S-transferase mu 2	*Gstm2*	2.01	0.85	2.96	1.61	5.54E-06
Glutathione S-transferase mu 3	*Gstm3*	3.02	0.87	4.54	1.33	4.5E-06
Glutathione S-transferase mu 4	*Gstm4*	1.50	0.84	2.37	1.28	9.418E-06
Glutathione S-transferase mu 5	*Gstm5*	1.35	0.88	1.79	1.14	5.69E-05
Glutathione S-transferase mu 6	*Gstm6*	1.67	0.79	2.41	1.16	1.25E-05
Glutathione S-transferase mu 7	*Gstm7*	1.57	0.81	2.30	1.16	1E-05
Glutathione S-transferase A1	*Gsta1*	1.72	0.86	2.70	1.40	0.00019
Glutathione S-transferase theta-1	*Gstt1*	1.08	1.12	1.30	0.92	0.22
Glutathione S-transferase theta-2	*Gstt2*	1.40	0.99	1.51	1.01	0.034
Fumarylacetoacetase	*Fah*	0.93	0.97	0.93	0.99	0.62
Catalase	*Cat*	1.16	1.02	1.44	1.08	0.038
Superoxide dismutase	*Sod2*	0.82	0.92	0.85	0.93	0.052
NAD(P)H dehydrogenase [quinone] 1	*Nqo1*	1.52	0.87	2.60	1.92	0.00027

INH, isoniazid; RIF, rifampicin; RIF/INH, rifampicin and isoniazid.

Supplementary Table S6. Relative change in proteins associated with wound healing and inflammation.

		hPXR			***mPxr*$^{-/-}$**	
**Protein**	**Gene**	**RIF/INH**	**INH**	**RIF**	**RIF/INH**	***p* Value**
Glutamine synthetase	*Glul*	0.85	1.03	0.72	1.17	0.16
Carbonic anhydrase 3	*Ca3*	1.23	1.14	1.18	1.19	0.81
Transforming growth factor-beta-induced protein ig-h3	*Tgfbi*	0.88	0.75	0.60	0.69	0.44
Protein-glutamine gamma-glutamyltransferase 2	*Tgm2*	1.12	0.81	1.19	0.88	0.0059
SPARC	*Sparc*	0.85	0.86	0.72	0.83	0.089
Collagen alpha-1(III) chain	*Col3a1*	1.94	0.34	0.28	0.35	0.056
Collagen alpha-1(IV) chain	*Col4a1*	1.27	0.86	0.83	0.90	0.0033
Collagen alpha-1(VI) chain	*Col6a1*	1.10	0.67	0.47	0.63	0.0091
Annexin A2	*Anxa2*	1.43	0.89	0.94	1.08	0.30
Annexin A5	*Anxa5*	1.56	0.98	1.57	1.22	0.032
Biglycan	*Bgn*	1.37	0.94	0.74	1.08	0.44
G1/S-specific cyclin-D1	*Ccnd1*	1.40	0.69	1.24	1.03	0.19
T-lymphocyte activation antigen CD86	*Cd86*	1.08	0.86	1.03	0.96	0.13

INH, isoniazid; RIF, rifampicin; RIF/INH, rifampicin and isoniazid.

Supplementary Table S7. Relative change in [Fe–S] cluster-containing proteins.

		hPXR			*mPxr*$^{-/-}$	
**Protein**	**Gene**	**RIF/INH**	**INH**	**RIF**	**RIF/INH**	***p* Value**
NADH dehydrogenase [ubiquinone] iron-sulfur protein 3	*Ndufs3*	0.94	1.09	1.01	1.09	0.74
NADH dehydrogenase [ubiquinone] iron-sulfur protein 2	*Ndufs2*	1.00	1.04	1.03	1.04	0.65
CDGSH iron-sulfur domain-containing protein 1	*Cisd1*	0.90	0.97	0.98	1.03	0.14
NADH dehydrogenase [ubiquinone] iron-sulfur protein 8	*Ndufs8*	0.91	0.96	0.82	0.75	0.0012
Succinate dehydrogenase [ubiquinone] iron-sulfur subunit	*Sdhb*	0.86	0.99	0.92	0.99	0.036
NADH dehydrogenase [ubiquinone] iron-sulfur protein 7	*Ndufs7*	1.00	1.14	1.09	1.20	0.012
NADH dehydrogenase [ubiquinone] iron-sulfur protein 4	*Ndufs4*	0.94	1.12	1.10	1.23	0.033
NFU1 iron-sulfur cluster scafrelative homolog	*Nfu1*	0.82	0.98	0.90	1.01	0.13
NFU1 iron-sulfur cluster scafrelative homolog, mitochondrial (Fragment)	*Nfu1*	0.82	0.99	0.87	0.96	0.059
Iron-sulfur protein NUBPL	*Nubpl*	1.27	1.77	1.37	0.91	0.067
Iron-sulfur cluster assembly enzyme	*Iscu*	0.95	1.01	0.96	0.98	0.84
NADH dehydrogenase [ubiquinone] iron-sulfur protein 6	*Ndufs6*	0.94	0.96	0.86	0.82	0.13
NADH dehydrogenase [ubiquinone] iron-sulfur protein 5	*Ndufs5*	0.87	0.97	0.91	1.03	0.22
CDGSH iron-sulfur domain-containing protein 2	*Cisd2*	0.90	0.88	0.89	0.82	0.94
Iron-responsive element-binding protein	*Ireb2*	1.19	1.21	1.42	1.28	0.039
2-oxoglutarate and iron-dependent oxygenase domain-containing protein 2	*Ogfod2*	1.11	0.97	1.15	0.99	0.51
Probable cytosolic iron-sulfur protein assembly protein	*Ciao1*	0.96	1.03	0.98	1.12	0.10
Iron-sulfur cluster assembly 2 homolog, mitochondrial	*Isca2*	0.90	0.91	1.13	1.02	0.20
CDGSH iron-sulfur domain-containing protein 3	*Cisd3*	0.84	0.88	1.02	1.07	0.060
2-oxoglutarate and iron-dependent oxygenase domain-containing protein 3	*Ogfod3*	1.03	0.98	0.91	0.87	0.25
Aldehyde oxidase 1	*Aox1*	1.30	0.82	1.65	1.48	0.0023
Xanthine dehydrogenase/oxidase	*Xdh*	0.70	0.70	0.75	0.98	0.0079

INH, isoniazid; RIF, rifampicin; RIF/INH, rifampicin and isoniazid.

Supplementary Table S8. Relative change in [Fe–S] cluster assembly machinery proteins.

		hPXR			*mPxr*$^{-/-}$	
**Protein**	**Gene**	**RIF/INH**	**INH**	**RIF**	**RIF/INH**	***p* Value**
Cysteine desulfurase	*Nfs1*	0.89	0.96	0.84	0.90	0.042
Isoform 2 of Low molecular weight phosphotyrosine protein phosphatase	*Acp1*	0.96	0.98	1.03	1.00	0.63
Frataxin, mitochondrial	*Fxn*	0.84	0.98	0.89	0.88	0.11
Ferredoxin-relative anticodon-binding domain-containing protein 1 homolog	*Fdxacb1*	1.06	0.96	1.09	1.15	0.94
Adrenodoxin	*Fdx1*	0.64	0.77	0.84	0.79	0.0016
NADPH:adrenodoxin oxidoreductase,	*Fdxr*	0.83	0.93	0.85	0.91	0.16
Stress-70 protein	*Hspa9*	1.07	1.07	0.94	1.05	0.32
Iron-sulfur cluster co-chaperone protein	*Hscb*	1.23	1.20	1.40	1.44	0.0056
GrpE protein homolog 2, mitochondrial	*Grpel2*	1.24	1.18	1.04	1.19	0.78
GrpE protein homolog 1	*Grpel1*	1.10	1.07	0.98	1.05	0.69
Glutaredoxin-related protein 5	*Glrx5*	1.60	1.69	1.24	1.39	0.078
Iron-sulfur cluster assembly 1 homolog	*Isca1*	0.76	0.68	0.66	0.81	0.0037
Iron-sulfur cluster assembly 2 homolog	*Isca2*	0.90	0.91	1.13	1.02	0.20
Putative transferase CAF17 homolog	*Iba57*	1.00	0.98	1.15	1.14	0.0090
NFU1 iron-sulfur cluster scafrelative homolog	*Nfu1*	0.82	0.98	0.90	1.01	0.13
NFU1 iron-sulfur cluster scafrelative homolog, mitochondrial (Fragment)	*Nfu1*	0.82	0.99	0.87	0.96	0.059
BolA-like protein 3	*Bola3*	0.89	0.91	1.12	1.03	0.012
Iron-sulfur protein	*Nubpl*	1.27	1.77	1.37	0.91	0.067
Cytosolic Fe-S cluster assembly factor	*Nubp1*	1.07	0.91	0.94	1.03	0.33
Cytosolic Fe-S cluster assembly factor	*Nubp2*	1.03	1.06	1.01	1.14	0.35
Anamorsin	*Ciapin1*	1.28	1.19	1.34	1.10	0.0095
BolA-like protein 2	*Bola2*	0.98	0.90	0.92	0.88	0.58
Cytosolic Fe-S cluster assembly factor	*Narfl*	1.00	1.01	1.06	1.01	0.65
Probable cytosolic iron-sulfur protein assembly protein	*Ciao1*	0.96	1.03	0.98	1.12	0.10
MMS19 nucleotide excision repair protein homolog	*Mms19*	0.96	0.96	0.97	1.07	0.22

INH, isoniazid; RIF, rifampicin; RIF/INH, rifampicin and isoniazid.

Supplementary Table S9. Relative change in proteins associated with vitamin B_6 metabolism.

		hPXR			***mPxr***$^{-/-}$	
**Protein**	**Gene**	**RIF/INH**	**INH**	**RIF**	**RIF/INH**	***p* Value**
Pyridoxal kinase	*Pdxk*	1.07	1.05	1.13	1.17	0.75
Pyridoxal phosphate phosphatase	*Pdxp*	1.49	0.95	2.18	1.34	0.00049
Pyridoxal-dependent decarboxylase domain-containing protein 1	*Pdxdc1*	0.97	1.03	1.01	1.02	0.84

INH, isoniazid; RIF, rifampicin; RIF/INH, rifampicin and isoniazid.

Supplementary Table S10. Relative change in proteins associated with homocysteine metabolism.

		hPXR			*mPxr*$^{-/-}$	
**Protein**	**Gene**	**RIF/INH**	**INH**	**RIF**	**RIF/INH**	***p* Value**
Methionine synthase	*Mtr*	1.08	1.02	1.26	1.11	0.045
Methylenetetrahydrofolate reductase	*Mthfr*	0.96	0.96	0.82	1.19	0.097
Cystathionine gamma-lyase	*Cth*	1.06	0.87	0.97	1.50	0.0081
Cystathionine beta-synthase	*Cbs*	0.62	0.81	0.62	0.76	0.00375
Betaine homocysteine S-methyltransferase 1	*Bhmt*	1.29	1.21	1.21	1.99	0.0229

INH, isoniazid; RIF, rifampicin; RIF/INH, rifampicin and isoniazid.

Supplementary Table S11. Relative change in proteins associated with tryptophan metabolism.

		hPXR			*mPxr*$^{-/-}$	
**Protein**	**Gene**	**RIF/INH**	**INH**	**RIF**	**RIF/INH**	***p* Value**
Tryptophan 2,3-dioxygenase	*Tdo2*	0.89	0.94	0.96	0.86	0.93
Kynurenine formamidase	*Afmid*	1.05	0.95	1.09	1.10	0.53
Indoleamine 2,3-dioxygenase 2	*Ido2*	0.93	0.79	0.81	1.00	0.052
3-hydroxyanthranilate 3,4-dioxygenase	*Haao*	0.91	0.99	0.91	1.14	0.0077
Kynurenine/alpha-aminoadipate aminotransferase	*Aadat*	0.62	0.74	0.56	0.79	0.0014
Kynurenine 3-monooxygenase	*Kmo*	0.82	0.91	0.78	1.11	0.016
Kynureninase	*Kynu*	1.00	0.98	1.09	1.15	0.057
Kynurenine--oxoglutarate transaminase	*Ccbl1*	0.82	0.83	0.79	0.71	0.12
2-amino-3-carboxymuconate-6-semialdehyde decarboxylase	*Acmsd*	0.83	0.57	0.80	0.72	0.09
Nicotinate phosphoribosyltransferase	*Naprt*	0.87	0.98	0.83	0.79	0.0062

INH, isoniazid; RIF, rifampicin; RIF/INH, rifampicin and isoniazid.

Supplementary Table S12. Protein changes by mouse strain.

Decreased			Increased		
***hPXR* only**	***mPxr*$^{-/-}$ only**	**Both**	***hPXR* only**	***mPxr*$^{-/-}$ only**	**Both**
ABCB7	ABCC8	AADAT	ABCC4	AADAC	AAMDC
ACAA2	ABHD1	AASS	ABHD1	ACAD9	ABCB1A
ACADSB	ACTN1	ABCA3	ACACA	ACNAT1	ABCC3
ACADVL	ACY1	ABCA6	ACAD11	ACNAT2	ABCD1
AGFG2	ADCK4	ABCC2	AKR1B10	ACP6	ABCD2
ALDH6A1	AKR1B1	ABCC6	AKR1B8	ADAL	ABCD3
ANKRD26	ALOX12	ABCG5	ALB	AGT	ABHD4
ARG1	ALOX12E	ABCG8	ALOX12	AHR	ABHD5
ARPC3	ANO6	ACSM5	ALPL	AK3	ABL1
ASPDH	ANPEP	AKR1C13	AMN1	AKR1A1	ACAA1A
ASS1	APRT	AKR1C19	AMY2	ALAS2	ACAA1B
ATG4B	ARHGAP1	ALCAM	ANXA7	ANKRD26	ACOT1
ATP11A	ARHGAP12	AMT	APOA1	ARFGAP1	ACOT2
ATP11C	ARHGAP27	AOX2	APOM	ARMC6	ACOT3
ATP5J2	ARHGEF40	AOX3	APP	ATP5I	ACOT4
BCO1	ARPC4	ARHGEF10L	APRT	BCKDK	ACOT6
BLVRA	ASPA	ARPC1B	ARL6IP6	BLOC1S1	ACOT8
C1QBP	ASPG	ASAH1	ASRGL1	BLOC1S5	ACOX2
CARS2	ATP6V1B2	ATP12A	ATG16L2	BOLA1	ACSS3
CCBL2	ATP6V1C1	ATP1A1	ATOX1	BRAT1	ACTR1B
CCDC58	ATP6V1E1	ATP1A3	BCAP31	CA5A	ACYP2
CCDC8	ATP6V1F	ATP1B1	BLVRB	CBX8	ADIPOR2
CDK2	BPIFA2	ATP8B1	BNIP1	CCDC174	AFM
CECR5	BSN	ATP8B3	BPIFA2	CCND3	AGPAT9
CHIL3	CA1	AVIL	CACYBP	CD81	AIFM2
CHST13	CA8	BC029214	CAT	CHCHD3	AKR1B7
CLCN4	CCDC124	BCL2L1	CBR1	CHCHD7	AKR1D1
CLCN5	CCDC85C	BDH2	CBR3	CLOCK	ALDH1A1
CMC4	CD1D1	CAD	CCDC124	COX16	ALDH1A2
CML1	CD74	CARKD	CCDC137	CTAGE5	ALDH1A3
CML2	CEL	CASP3	CD5L	CTH	ALDH1A7
COX6A1	CELA2A	CBS	CD74	CYB5B	ALDH3A2
COX6B1	CES2A	CCDC53	CDA	CYHR1	ANAPC4
CPS1	CES2B	CCDC93	CEL	CYP2C40	ANGPTL3
CYB5R1	CES2G	CCT8L1	CELA2A	CYP2C67	ANO10
CYP2C40	CIRBP	CD163	CES1D	CYP2C68	ANXA5
CYP2C67	CLEC3B	CD177	CES2A	CYP2C69	AOX1

Decreased			Increased		
***hPXR* only**	***mPxr*$^{-/-}$ only**	**Both**	***hPXR* only**	***mPxr*$^{-/-}$ only**	**Both**
CYP2C68	COL5A2	CD300LD	CES2B	D2WSU81E	APOA2
CYP2C69	COL6A1	CDH1	CES5A	DCTN3	APOA4
CYP4F14	COL6A2	CEBPB	CHMP5	DCTN4	APOB
DBT	COL6A3	CES3B	CHP1	DEF8	APOC1
DCPS	COL6A5	CHSY3	CIAPIN1	DOLPP1	APOC4
DDI2	CPA1	CNP	CMAS	DPYD	APOE
DHRS11	CRTAP	COQ3	CNIH4	DTNBP1	APOF
EBP	CTRB1	COQ5	COL1A1	DTX3L	APOH
ERBB2	CTSA	COQ6	COL1A2	EBP	ARL2
ETFB	CYFIP2	COQ7	COL4A1	EEF1E1	ARPP19
FAHD1	CYP2C29	CPNE2	COL4A2	EIF2AK4	ATP2C1
FAN1	CYP2C38	CTSF	COL5A2	ESRP1	ATP9A
FOXRED1	CYP2C39	CYP1A1	COL6A5	FAM126A	AURKAIP1
GALT	CYP2C55	CYP1A2	CP	FAM169B	BC027231
GATM	DFFB	CYP27A1	CPA1	FAM173B	BET1
GCSH	DGCR6	CYP2D26	CPN2	FAM210A	BHMT
GJB1	DHPS	CYP2F2	CREB3L3	FBXL17	BLOC1S4
GLS2	DHRS9	CYP2U1	CREG1	FBXW5	BSDC1
GLUD1	DIO1	CYP4B1	CTRB1	FCN1	C4BPA
GNE	DKC1	DEGS1	CYP2A4	FHIT	CAPG
GSDMDC1	DLC1	DNAJA4	CYP2A5	FOCAD	CCDC86
HDAC6	DLG2	DYNC1I1	CYP2B10	FV4	CCDC91
HIP1R	DNAJC10	EGFR	CYP2C29	G6PC	CD36
HMGN5	DNM2	ENPP1	CYP2C38	GANC	CDC34
INSC	ECE1	EPB4	CYP2C39	GATM	CDK5RAP1
ISYNA1	EML4	EPB41L5	CYP2C55	GBAS	CDK6
KEG1	EPN2	ERP44	CYP3A59	GCH1	CDKN2C
KIFAP3	FAM69A	FABP5	D8ERTD738E	GIMD1	CENPV
KMO	FARP1	FAM210B	DAPK2	GIPC1	CES1
LASP1	FAT3	FDX1	DDAH1	GJB2	CES1B
LDHA	FBXW9	FECH	DERL1	GLB1	CES1C
LETM1	FERMT3	FEZ2	DGKE	GLB1L	CGRRF1
LETMD1	FHOD1	FGFR1	DHX37	GLDC	CHCHD6
MCCC1	FUOM	FGFR2	DLAT	GLRX2	CIDEB
MCCC2	G6PDX	FGFR3	DMPK	GLS2	CMBL
MMGT1	GALNT11	FRRS1	DNAJA2	GLYCTK	COA3
MOCS1	GFPT1	FSCN1	DUSP3	GM20441	CPPED1
MRRF	GLOD4	FTL1	DYNC1LI2	GNMT	CRAT

Decreased			Increased		
***hPXR* only**	***mPxr$^{-/-}$* only**	**Both**	***hPXR* only**	***mPxr$^{-/-}$* only**	**Both**
MT-ATP6	GM11992	GBA	ECHDC1	GPHN	CRCP
MT-CO3	GM28048	GDPGP1	EMG1	GULO	CRP
MTFR1L	GMFB	GM14434	F12	H2-T23	CUEDC2
MTND3	GP5	GM17296	F13B	HAO1	CYB5A
NADK2	GPI	GM4450	FAM192A	HIRA	CYB5R3
NAT8	GRTP1	GMPR	FBXW9	HSD17B10	CYP2E1
NDUFAF2	GSTA4	GPR155	FGB	HUS1	CYP3A11
NDUFAF5	GSTO2	HAL	FHAD1	ISOC2B	CYP3A16
NDUFAF6	H13	HDHD3	FKBP1A	ITFG1	CYP3A25
NINJ1	H2-AB1	HGSNAT	FMNL2	KCNH8	CYP3A41A
NME3	H2-DMB1	HM13	FN1	LARS2	CYP3A44
NMNAT3	H2-EB1	HMGB3	FUT8	LGALS8	CYP3A57
NNMT	HMHA1	HOGA1	GALE	LIN7C	CYP4A10
NOSTRIN	HNF1A	HPSE	GP1BA	LNP	CYP4A14
NR5A2	HNRNPD	HSD17B2	GP5	LRRC20	CYP4A31
OTC	HSP90B1	HSD3B4	GP9	LRSAM1	CYP4A32
PACSIN3	HYAL1	HSD3B5	GSN	MAPKAPK3	CYP8B1
PAH	ICMT	ICAM1	GSTA4	MAR1	D17WSU92E
PAK7	IDUA	IGJ	GSTM5	MAST2	D2HGDH
PCBD2	IGHA	ISCA1	GSTT2	MAT2A	DCTN1
PCDH1	IGHV1-4	IVD	H2-AB1	MCC	DDX52
PLXNB1	IGHV1-62-2	JAKMIP3	H2-DMB1	MCEE	DECR2
PNKD	IGHV5-4	KBTBD4	H2-EB1	METTL17	DENND1B
POLG2	IGHV7-2	KIAA0195	HAUS3	MIA2	DHCR24
POLR2H	IGLC2	LIPO1	HGFAC	MIPEP	DHDDS
PPT1	IMPA1	LPIN1	HNRNPM	MKNK1	DHRS7B
PRODH	INMT	LRP1	HOPX	MME	DLG5
PRODH2	INPP5F	LRP1B	HSPB1	MPDZ	DNAH7B
PTDSS1	IPO9	LTF	ICA	MPST	DNAJB2
PTMS	ITGA7	MANBA	IFI27L2B	MRPL1	EEPD1
PTPMT1	KCNN2	MCU	IFIT1	MRPL21	EGLN1
PTPRS	KDM1A	MICU1	IGHV1-9	MRPL40	EHHADH
RING1	KDR	MIER1	IGHV7-2	MRPS11	EIF4ENIF1
S100A1	KIF5B	MMP9	IGLC2	MRPS17	ELOVL6
SARS2	KLC2	MOXD1	IGSF5	MRPS22	ENSA
SLC1A2	KLC4	MT-CO1	ITGA2B	MRPS24	ENTPD5
SLC22A1	LRRC32	MTHFD2L	ITIH1	MRPS30	EPHX1
SLC25A12	LRRC47	MYO1B	KIAA0100	MRPS36	ERMP1

Decreased			Increased		
***hPXR* only**	***mPxr*$^{-/-}$ only**	**Both**	***hPXR* only**	***mPxr*$^{-/-}$ only**	**Both**
SLC25A15	LSR	NAAA	KIF21A	MRPS7	F10
SLC25A25	LTA4H	NAGA	KLHDC10	MRPS9	F2
SLC25A45	MAN2B1	NAGLT1A	KLKB1	MSRB2	FAAP100
SLC25A48	MAN2B2	NAGLU	KNG1	MTAP	FAM175B
SLC25A51	MAP1S	NAPSA	KNG2	MTCH1	FAM185A
SLC26A1	MAP3K15	NASP	LGALS3	MTND3	FAM195A
SLC35A3	MARK4	NDST1	LPIN3	MTX1	FAM32A
SLC8A2	MBNL1	NFIX	LRRC32	MYEF2	FAM73B
SLCO1A1	MOV10	NGP	LTBP1	NAA25	FDFT1
SLCO2B1	MPP7	NPC1	MAPRE3	NAGS	FER1L6
SNAP25	MRI1	NPHP3	MARCH6	NANP	FITM2
SRRM2	MST1	PALD1	MGLL	NAV2	FKBP8
STX11	MX1	PCBP4	MICAL2	NDRG2	FKBPL
SUCLA2	MX2	PCK2	MMRN1	NDUFA5	FMO1
SULT2A4	MYO1A	PGAP1	MST1	NDUFA7	FMO5
TAT	MYT1L	PGLYRP1	MSTO1	NDUFAF7	FMO9
THEM4	NAPRT	PGPEP1	MVP	NDUFB11	FOXP1
TIMM10B	NCBP1	PHLDA1	MYO19	NDUFB9	GALK1
TIMM44	NDRG1	PLA2G12B	MYO1A	NDUFS1	GDE1
TMEM11	NDUFB5	PLBD1	MYO5B	NDUFS4	GGCT
TMEM126A	NDUFS8	PLBD2	NAA20	NDUFS7	GM10639
TMEM201	NOS3	PNP	NADK	NMNAT3	GM4952
TMEM261	NT5C3B	PPOX	NAGK	NT5DC1	GM4978
TRAPPC5	NUDT16	PTGES2	NCEH1	NUDT2	GNPAT
TSPAN31	OSBPL1A	PTGS2	NCF1	OMA1	GPAM
TSPAN33	OSGEP	PTPRF	NCLN	PACS2	GPT2
TTC23L	OVCA2	PYROXD2	NEB	PANK1	GPX4
TTN	PAPSS1	RAB24	NEBL	PDE4C	GPX7
TVP23B	PARP3	RASSF3	NMT2	PEBP1	GRIN3B
UQCRC1	PEPD	RBM33	NOMO1	PEX12	GSTA1
UQCRH	PET117	RER1	NOS2	PI4KA	GSTM1
URAD	PF4	RETN	NRDE2	PLEKHA3	GSTM2
USP46	PFAS	RETNLG	PEX11G	PLXNB1	GSTM3
VDAC3	PFKM	RNASET2	PF4	PPCDC	GSTM4
XDH	PGAM5	S100A9	PGRMC2	PPFIBP2	GSTM6
XPNPEP3	PHF6	SDC4	PIGX	PRKACA	GSTM7
ZBTB7B	PHYKPL	SDHD	PLA2G7	PRKCZ	GSTT3
ZDHHC20	PLCB1	SDSL	PLEK	PRKD2	GZF1

Decreased			Increased		
***hPXR* only**	***mPxr*$^{-/-}$ only**	**Both**	***hPXR* only**	***mPxr*$^{-/-}$ only**	**Both**
ZNF830	PLCB3	SELENBP1	PLEKHA1	PRKRA	H1F0
	PLSCR1	SELENBP2	PNLIP	PTPMT1	H2-Q10
	PNKP	SENP6	PNLIPRP1	PXMP2	H3F3A
	PNLIP	SIAE	POLR1E	RBM17	HIST1H1A
	PODXL	SLC12A9	POR	RDH10	HIST1H1B
	POLR2I	SLC17A3	PPFIBP1	RDH5	HIST1H1C
	PPFIBP1	SLC22A7	PRKCA	RMDN2	HIST1H1E
	PPIC	SLC25A37	PROZ	RNF14	HIST1H3A
	PPP1R37	SLC29A1	PSAT1	RPP25L	HIST1H3B
	PRKAB2	SLC2A4	PSMD5	RRP1B	HMGCS1
	PSIP1	SLC30A1	PXMP4	SAMD4B	HMGCS2
	PSME1	SLC35A2	PZP	SEC16A	HPGD
	PTMA	SLC39A14	REEP5	SERAC1	HSCB
	PYGB	SLC6A12	REEP6	SERPING1	HSD17B12
	R3HCC1	SMIM12	RFX5	SIRT2	HSD17B4
	R3HDM2	SMPD1	RGL2	SLC1A2	HSD17B7
	RAB11FIP1	ST3GAL4	RIN2	SLC25A35	HTATIP2
	RAB13	STARD3	RNF185	SLC35A3	IGHV5-12
	RAB3A	STEAP3	ROBO1	SLCO1A1	IL1RAP
	RAB3C	STOML2	RPF2	STARD10	IMMT
	RAD21	STXBP5L	RPIA	SUGCT	IREB2
	RELN	TAF12	RPL18	SULT5A1	ITPKB
	RGS16	TCIRG1	RPL22L1	TACO1	KANSL3
	RIOK3	TGM1	RPS25	TCAIM	KDSR
	RNF181	TMEM14C	SAV1	TM7SF2	LAMA1
	ROBO1	TMEM167B	SDC1	TMEM102	LGALS1
	RP2	TMEM30A	SERPINA1B	TMEM135	LHPP
	RPS16	TPST1	SERPINA1D	TMEM2	MAN2C1
	RTKN	TRAM1	SERPINA6	TMEM260	MAPKAPK5
	RUNDC1	TSC22D1	SERPINB1A	TMX2	MASP2
	RWDD1	TSC22D2	SERPINB1C	TSR2	MAT1A
	SAG	TSC22D3	SERPINB6B	TTC39B	ME1
	SART3	TSC22D4	SERPINC1	TTC39C	METTL7B
	SCARB2	TTC19	SERPINF1	TXN	METTL9
	SDC1	UBL3	SERPINF2	UGT3A1	MGME1
	SEPHS1	UBXN7	SLC23A2	UGT3A2	MGMT
	SERPINB1A	UQCC1	SLCO1A4	USP38	MGST1
	SETD6	UQCC2	SLPI	ZFP219	MMP14

	Decreased			Increased	
***hPXR* only**	***mPxr*$^{-/-}$ only**	**Both**	***hPXR* only**	***mPxr*$^{-/-}$ only**	**Both**
	SH3GLB1	UQCRQ	SMAD2	ZNF706	MRPL11
	SIRT6	VAMP5	SMTN		MRPL30
	SLC2A3	VPS37C	SNAP29		MRPL35
	SLPI	WDR20	SPAST		MRPL47
	SMARCA2		SPP2		MRPL57
	SMC1A		SRCIN1		MRPS12
	SMS		ST3GAL5		MRPS15
	SNRPB2		SULT2A1		MRPS18B
	SNW1		SYNE3		MRPS21
	SOAT2		SYNJ2		MRPS25
	SPAST		SYNPO		MRPS35
	SPTBN2		TCN2		MTERF1A
	SRCIN1		TDRD7		MTERF2
	SRSF10		TDRKH		MTG1
	SSR3		TEP1		N6AMT2
	STAT2		THBS1		NBEAL1
	TIMP3		TIMM8B		NEU1
	TMA7		TIMP3		NKTR
	TMEM51		TMA7		NOL3
	TMEM87A		TMEM109		NPRL3
	TNKS1BP1		TMEM259		NQO1
	TOM1L2		TMEM51		NSDHL
	TPM4		TMX1		NUDT12
	TPRKB		TNFSF10		OAT
	TRAPPC1		TPM4		OBFC1
	TRIM28		TRUB2		ODR4
	TRMT11		TTC7		OSBPL3
	TRP53RK		TXNDC15		P4HTM
	TRY10		UBASH3B		PAFAH2
	TUBGCP3		UGGT2		PANK2
	U2AF2		UGT1A1		PCTP
	UBA5		UGT1A10		PDK1
	UBE2H		UGT1A7C		PDK2
	UBE2M		UGT2B34		PDXP
	UBIAD1		UGT2B35		PEO1
	UFC1		UGT2B36		PEX10
	ZNF511		UPP1		PEX11A
	ZWINT		USE1		PEX13

Decreased			Increased		
***hPXR* only**	***mPxr*$^{-/-}$ only**	**Both**	***hPXR* only**	***mPxr*$^{-/-}$ only**	**Both**
			VNN3		PEX14
			WDR77		PEX16
			ZCCHC11		PEX19
			ZFPL1		PEX2
					PEX26
					PEX3
					PEX6
					PGD
					PGLYRP2
					PGS1
					PITRM1
					PKHD1
					PLA2G6
					PLIN2
					PLIN4
					PLTP
					PNPLA2
					PNPLA3
					PNPLA8
					POMP
					PRUNE
					PSMG3
					PTK2B
					PTS
					PUSL1
					RAB30
					RAD1
					RARRES1
					RBFA
					RBM8
					RBM8A
					RBP4
					RDH16
					RDH9
					REXO2
					RPL14
					RPL24
					RPL28

Decreased			Increased		
***hPXR* only**	***mPxr$^{-/-}$* only**	**Both**	***hPXR* only**	***mPxr$^{-/-}$* only**	**Both**
					RPL35
					RPL4
					RPL6
					RPL7
					RPL7A
					RPL8
					RPS6KA1
					RRP36
					RSL1D1
					RTFDC1
					RTP4
					SDHAF1
					SECISBP2L
					SERF2
					SERPINA7
					SHCBP1L
					SLAIN2
					SLC22A18
					SLC47A1
					SRXN1
					STAU2
					STRADB
					STS
					SVIP
					SYAP1
					TBCE
					TBCEL
					TECR
					TEX2
					THRSP
					TKT
					TMCC3
					TMEM120A
					TMEM147
					TMEM205
					TMEM263
					TMLHE
					TMPRSS11B

Decreased			Increased		
***hPXR* only**	***mPxr*$^{-/-}$ only**	**Both**	***hPXR* only**	***mPxr*$^{-/-}$ only**	**Both**
					TOP1MT
					TPD52
					TRAK2
					TRIAP1
					TSSC4
					TTPA
					TTR
					TYW1
					UAP1L1
					UBE2L6
					UBXN4
					UCK1
					UCKL1
					UGDH
					UGT1A5
					UGT1A9
					UROS
					USP40
					USP45
					VNN1
					WDR34
					WDR73
					WWOX
					ZBTB21
					ZGPAT
					ZNF22
					ZNF804A
					ZNHIT2

Supplementary Table S13. Proteins decreased by treatment in *hPXR* mice.

INH only	RIF only	RIF/INH	INH and RIF only, but not RIF/INH	RIF only and RIF/INH, but not INH only	INH only and RIF/INH, but not RIF only	INH only, RIF only, and RIF/INH
ABHD4	AARSD1	ACADVL	ABHD1	ABCA6	ABCC2	AADAT
ACYP2	ABCC8	ALDH6A1	AMY2	ABCB7	AKR1C13	AASS
AGPAT1	ABCC9	AMT	ARHGAP27	ABCC6	AKR1C19	ABCA3
AKR1B8	ACP6	ANKRD26	ARL6	ACAA2	COQ7	ABCG5
ALOX12E	ACTN1	ARPC3	CEL	ACADSB	CYP1A1	ABCG8
ANPEP	ACTN4	ASPDH	CELA2A	ACSM5	CYP1A2	AOX2
AOX1	ADCK2	ATG4B	COL1A1	AGFG2	CYP2D26	AOX3
ARHGDIA	AK2	BDH2	COL1A2	ALCAM	CYP4B1	ARPC1B
ATP6V1E1	AKR1C21	C1QBP	COL5A2	ARG1	FECH	ASAH1
CD1D1	AKR1C6	CARKD	COL6A1	ARHGEF10L	GATM	ASS1
CES2C	ALAS2	CASP3	COL6A2	ATP11A	KIAA0195	ATP8B3
CHID1	ANO6	CHST13	COL6A3	ATP11C	PNKD	AVIL
CREG1	AP2A1	COQ3	COL6A5	ATP12A	PPOX	BC029214
CRTAP	ARFGAP2	COQ5	CPA1	ATP1A1	PRODH2	BCL2L1
CYP2A12	ATP13A1	COQ6	CTRB1	ATP1A3	PYROXD2	CAD
CYP2A22	BRD3	COX6A1	CTSA	ATP1B1	SLC6A12	CBS
CYP2A4	BRMS1	COX6B1	DGCR6	ATP5J2	TRAPPC5	CCBL2
CYP2A5	CA5A	DHRS11	DHRS9	ATP8B1	TTC19	CCDC53
CYP2C55	CA8	FOXRED1	FAT3	BCO1		CCDC8
DDC	CAMK1D	GALT	GM11992	BLVRA		CD163
DLG2	CCDC51	HMGB3	GRTP1	CARS2		CD177
DNAJC10	CCDC85B	JAKMIP3	GSTO2	CCDC58		CD300LD
DOPEY2	CCDC85C	MT-CO3	ICMT	CCDC93		CDH1
GFPT1	CDKN2AIP	MTND3	IGHV1-4	CCT8L1		CEBPB
GJB2	CLCN3	NADK2	IGHV1-62-2	CDK2		CES3B
GM10639	CLEC3B	NDUFAF2	IGHV5-4	CECR5		CHSY3
GSTA4	CMAH	NDUFAF5	ITGA7	CHIL3		CML2
GSTM1	CNPY4	NDUFAF6	KCTD12	CLCN4		CPS1
GSTM2	COL4A1	NOSTRIN	NAB2	CLCN5		CTSF
GSTM4	COPG1	NPHP3	PET117	CMC4		CYB5R1
GSTM6	COX7A2	PNP	PNLIP	CML1		CYP27A1
GSTM7	CSTF2T	POLR2H	PODXL	CNP		CYP2C40
H2-AB1	CTAGE5	PTPMT1	RNF25	CPNE2		CYP2C68
H2-DMB1	CTNNBL1	RASSF3	RP2	CYP2C67		CYP2C69
IDUA	CYFIP2	S100A1	RWDD1	DBT		CYP2F2
IFI27L2B	DAB2	SLC25A37	SAG	DCPS		CYP2U1
IMPACT	DDX39A	SLC25A45	SLC43A3	DDI2		CYP4F14

INH only	RIF only	RIF/INH	INH and RIF only, but not RIF/INH	RIF only and RIF/INH, but not INH only	INH only and RIF/INH, but not RIF only	INH only, RIF only, and RIF/INH
ITPKB	DDX39B	SLC25A51	SULT5A1	DNAJA4		DEGS1
LGALS3	DHODH	SLC35A2	TMEM106B	EBP		DYNC1I1
MAN2B2	DHX38	SLCO2B1	TMEM41A	EGFR		ERP44
MYT1L	DHX9	SNAP25	UBE2H	ENPP1		FAM210B
NCF1	DKC1	TIMM10B		EPB4		FDX1
NDRG1	DLC1	TMEM201		EPB41L5		FEZ2
NOL3	DNM2	TTN		ERBB2		FGFR1
PAK1	DNM3	TVP23B		ETFB		FRRS1
PEPD	DNPH1	UQCRC1		FABP5		FSCN1
PIR	EAF1	UQCRH		FAHD1		FTL1
PLIN4	ECE1	VPS37C		FAN1		GBA
PON1	EMC4			FGFR2		GDPGP1
RAB3A	EMC6			FGFR3		GJB1
RNF185	EPB41L2			GCSH		GM14434
SHCBP1L	EPHA3			GLS2		GM17296
TGM2	EPN2			GLUD1		GPR155
TMEM51	FAM210A			GM4450		HAL
TMEM87A	FAM69A			GMPR		HDHD3
TRAK2	FERMT2			GNE		HGSNAT
TRAPPC1	FHOD1			GSDMDC1		HIP1R
UAP1L1	FOXO1			HDAC6		HM13
UGDH	FUOM			HMGN5		HOGA1
UGT2B35	FV4			HSD3B4		HPSE
UGT2B36	GCC1			HSD3B5		HSD17B2
ZNF804A	GFRA1			KEG1		ICAM1
	GIMAP4			KIFAP3		IGJ
	GLDC			KMO		INSC
	GM28048			LASP1		ISCA1
	GOT2			LDHA		ISYNA1
	GPRIN3			LETM1		IVD
	HDAC1			LETMD1		KBTBD4
	HMBOX1			LRP1		LIPO1
	HNF1A			LRP1B		LPIN1
	HNRNPD			MCCC1		LTF
	HYAL1			MCCC2		MANBA
	IGHA			MICU1		MCU
	IGHV1-9			MOCS1		MIER1
	IGHV7-2			MRRF		MMGT1

INH only	RIF only	RIF/INH	INH and RIF only, but not RIF/INH	RIF only and RIF/INH, but not INH only	INH only and RIF/INH, but not RIF only	INH only, RIF only, and RIF/INH
	IGLC2			MT-ATP6		MMP9
	IIGP1			MT-CO1		MOXD1
	INPP4A			MTFR1L		MTHFD2L
	IPO9			MYO1B		NAAA
	ITPR2			NAGLT1A		NAGA
	KCNN2			NASP		NAGLU
	KHNYN			NDST1		NAPSA
	L3MBTL3			NFIX		NAT8
	LNPEP			NME3		NGP
	LSR			NMNAT3		NINJ1
	LYRM4			NPC1		NNMT
	MACROD1			NR5A2		PAH
	MAP2K6			OTC		PCBP4
	MAP3K15			PACSIN3		PCK2
	MARK4			PAK7		PGLYRP1
	MBNL1			PALD1		PGPEP1
	MCC			PCBD2		PLA2G12B
	MED6			PCDH1		PLBD1
	MOGS			PGAP1		PLBD2
	MTFP1			PHLDA1		PLXNB1
	MTIF2			POLG2		PPT1
	MX1			PRODH		PTGS2
	MX2			PTDSS1		RER1
	NAGS			PTGES2		RETN
	NAPRT			PTMS		RETNLG
	NCBP1			PTPRF		RNASET2
	NDUFB5			PTPRS		S100A9
	NDUFS8			RAB24		SDC4
	NFS1			RBM33		SDSL
	NOL10			RING1		SELENBP1
	NONO			SARS2		SELENBP2
	NRF1			SDHD		SENP6
	NSRP1			SLC12A9		SIAE
	OPTN			SLC1A2		SLC17A3
	PAPD5			SLC22A1		SLC26A1
	PDCD2			SLC22A7		SLC39A14
	PDGFRA			SLC25A12		SMIM12
	PDS5B			SLC25A15		SMPD1

INH only	RIF only	RIF/INH	INH and RIF only, but not RIF/INH	RIF only and RIF/INH, but not INH only	INH only and RIF/INH, but not RIF only	INH only, RIF only, and RIF/INH
	PFAS			SLC25A25		STARD3
	PHF6			SLC25A48		STEAP3
	PLCB3			SLC29A1		STXBP5L
	PNKP			SLC2A4		TAF12
	POLR2I			SLC30A1		TAT
	PPIG			SLC35A3		TCIRG1
	PREX2			SLC8A2		TGM1
	PRKRIR			SLCO1A1		TMEM14C
	PROX1			SRRM2		TMEM167B
	PRRC2C			ST3GAL4		TRAM1
	PSIP1			STOML2		TSPAN33
	PSPH			STX11		UBL3
	PTMA			SUCLA2		VAMP5
	RAB11FIP1			SULT2A4		WDR20
	RAB13			THEM4		XDH
	RAB3IP			TIMM44		
	RAD21			TMEM11		
	RBBP4			TMEM126A		
	RBBP7			TMEM261		
	RELN			TMEM30A		
	RFWD2			TPST1		
	RNF20			TSC22D1		
	RNF213			TSC22D2		
	RRBP1			TSC22D3		
	SART1			TSC22D4		
	SART3			TSPAN31		
	SEC16A			TTC23L		
	SEC31A			UBXN7		
	SEPHS1			UQCC1		
	SERPING1			UQCC2		
	SF1			UQCRQ		
	SF3B5			URAD		
	SFPQ			USP46		
	SH3GLB1			VDAC3		
	SIAH1A			XPNPEP3		
	SIN3A			ZBTB7B		
	SLC25A35			ZDHHC20		
	SLC35A1			ZNF830		

INH only	RIF only	RIF/INH	INH and RIF only, but not RIF/INH	RIF only and RIF/INH, but not INH only	INH only and RIF/INH, but not RIF only	INH only, RIF only, and RIF/INH
	SLC9A3R2					
	SMARCA2					
	SMC1A					
	SMC6					
	SMEK2					
	SNRPA1					
	SNRPB2					
	SNW1					
	SNX29					
	SOAT2					
	SPTBN2					
	SRA1					
	SRCIN1					
	SRRM1					
	SSR3					
	SSRP1					
	ST3GAL3					
	STAM					
	STAT6					
	SUB1					
	SUDS3					
	SUPT16					
	TARSL2					
	TBK1					
	TLE1					
	TOM1L2					
	TRIP4					
	TRY10					
	TTC39B					
	TTC39C					
	U2AF2					
	UBE2M					
	VAMP8					
	WDR33					
	WDR55					
	XPO5					
	YARS2					
	ZNF592					

INH only	RIF only	RIF/INH	INH and RIF only, but not RIF/INH	RIF only and RIF/INH, but not INH only	INH only and RIF/INH, but not RIF only	INH only, RIF only, and RIF/INH
	ZNF687					

INH, isoniazid; RIF, rifampicin; RIF/INH, rifampicin and isoniazid combination treatment.

Supplementary Table S14. Proteins increased by treatment in *hPXR* mice.

INH only	RIF only	RIF/INH	INH and RIF only, but not RIF/INH	RIF only and RIF/INH, but not INH only	INH only and RIF/INH, but not RIF only	INH only, RIF only, and RIF/INH
ACAT1	ABHD3	ABHD1	BCKDK	ABCB1A	APOA1	AAMDC
ALAS2	ACAD12	ALOX12	CD81	ABCC3	APOA4	ABCD3
BCL9L	ACTR10	AMY2	CHCHD2	ABCC4	AURKAIP1	ABHD5
CBX8	AGPAT6	ANAPC4	COX16	ABCD1	CYP2E1	ABL1
CCDC174	AKR1A1	APOM	CRBN	ABCD2	EIF4ENIF1	ACAA1A
CHCHD7	AKR1C19	ARL6IP6	DEF8	ABHD4	HNRNPM	ACAA1B
CLOCK	ANPEP	BCAP31	EIF4H	ACAD11	IGHV5-12	ACACA
DNPH1	APIP	C4BPA	G6PDX	ACOT8	MMP14	ACOT1
DOLPP1	APPBP2	CCDC124	GIMD1	ACOX2	MRPL11	ACOT2
EIF1AD	ASPA	CCDC137	GM1840	ACTR1B	MRPL30	ACOT3
ESF1	ATG7	CD74	GPI	ACYP2	MRPL35	ACOT4
FABP5	ATP7B	CEL	HNMT	AFM	MRPL47	ACOT6
FBXL17	ATXN10	CELA2A	HUS1	AKR1B10	MRPL57	ACSS3
FLT1	BABAM1	CNIH4	IQCB1	AKR1B7	MRPS15	ADIPOR2
G6PC	BAG6	COL1A1	KCNH8	AKR1B8	MRPS18B	AGPAT9
GLDC	BLOC1S5	COL1A2	KHK	AKR1D1	MRPS25	AIFM2
GLRX2	BOLA1	COL4A1	LRRC28	ALDH1A1	MRPS35	ALB
HAO1	C2CD2	COL4A2	MAT2A	ALDH1A2	PKHD1	ALDH3A2
IGF2BP2	CBLB	COL5A2	MTMR9	ALDH1A3	RBFA	APOA2
MAK16	CCND3	COL6A5	NDUFAF7	ALDH1A7	RFX5	APOB
MARC1	CCT6B	CPA1	OMA1	ALPL	SERF2	APOC1
MRPL1	CCT8	CPN2	PANK1	AMN1	SPAST	APOE
MRPL21	CDC42	CTRB1	PKLR	ANGPTL3	TMEM147	APOF
MRPL40	CES2C	DENND1B	PSPC1	ANO10		APOH
MRPS11	CES2G	EGLN1	RAB11FIP2	ANXA5		ARPP19
MRPS17	CHDH	F13B	RBM17	ANXA7		ATP2C1
MRPS24	CHID1	FGB	RCBTB1	AOX1		ATP9A
MRPS30	CLPTM1L	FMNL2	RGS16	APOC4		BC027231
MRPS7	COG8	FMO5	SGPP1	APP		BHMT
MRPS9	CYB5B	FN1	SUGCT	APRT		BPIFA2
MSH3	CYHR1	GDE1	TDP2	ARL2		CCDC86
MT-CO3	CYP1A1	GSN	ZBED5	ASRGL1		CIAPIN1
MTND3	CYP1A2	H2-AB1	ZFP219	ATG16L2		CIDEB
MTND5	CYP2A12	H2-DMB1		ATOX1		COA3
MTX1	CYP2A22	H2-EB1		BET1		CPPED1
MYEF2	DCTN3	IGHV1-9		BLOC1S4		CRAT
NANP	DCTN4	IGHV7-2		BLVRB		CYP3A11

INH only	RIF only	RIF/INH	INH and RIF only, but not RIF/INH	RIF only and RIF/INH, but not INH only	INH only and RIF/INH, but not RIF only	INH only, RIF only, and RIF/INH
NDUFB11	DFFB	IGLC2		BNIP1		CYP3A16
OPTN	DOPEY2	IMMT		BSDC1		CYP4A10
PDE4C	DTNBP1	ITIH1		CACYBP		CYP4A14
RDH10	DUS2	KLKB1		CAPG		CYP4A31
RMDN2	DVL3	KNG2		CAT		CYP4A32
RPL7L1	EEF1E1	LGALS3		CBR1		CYP8B1
RPP25L	EIF2AK4	LRRC32		CBR3		D17WSU92E
S100A1	ERLEC1	LTBP1		CCDC91		D2HGDH
SAP30L	ESRP1	MAPKAPK5		CD36		D8ERTD738E
SARNP	FAM126A	MRPS21		CD5L		DAPK2
SCARB2	FAM169B	MST1		CDA		DDAH1
SLC10A1	FAM63B	MTERF1A		CDC34		DDX52
SLC29A1	FBXO4	MYO1A		CDK5RAP1		DHDDS
SLC2A4	FBXW5	NCLN		CDK6		DHRS7B
SLC35A3	FHIT	NEU1		CDKN2C		DLAT
SLC44A1	FOCAD	NOMO1		CENPV		DLG5
TACO1	GANC	NOS2		CES1		EHHADH
TJP3	GET4	PLA2G7		CES1B		ELOVL6
TNKS1BP1	GFPT1	PNLIP		CES1C		EMG1
ZBTB45	GINS4	PNLIPRP1		CES1D		ENSA
	GLYCTK	PPFIBP1		CES2A		FAAP100
	GM20441	RAD1		CES2B		FAM185A
	GMFB	RGL2		CES5A		FAM192A
	GSR	RNF185		CGRRF1		FAM195A
	HINT3	RPL18		CHCHD6		FAM32A
	IBA57	RPS25		CHMP5		FAM73B
	IMPACT	SERPINA1B		CHP1		FER1L6
	INMT	SERPINA1D		CMAS		FITM2
	ISOC2B	SLC23A2		CMBL		FKBP8
	KIF16B	SRCIN1		CP		FKBPL
	LNP	SYNE3		CRCP		FMO1
	LRRC20	TECR		CREB3L3		FOXP1
	MAST2	TIMM8B		CREG1		GM4952
	MBLAC2	TPM4		CRP		GM4978
	MCEE	UBE2L6		CUEDC2		GP1BA
	MIEN1	UGGT2		CYB5A		GP9
	MLKL	UGT2B36		CYB5R3		GPAM
	MME	ZGPAT		CYP2A4		GPT2

INH only	RIF only	RIF/INH	INH and RIF only, but not RIF/INH	RIF only and RIF/INH, but not INH only	INH only and RIF/INH, but not RIF only	INH only, RIF only, and RIF/INH
	MOCOS			CYP2A5		GRIN3B
	MROH1			CYP2B10		GZF1
	MSRA			CYP2C29		H1F0
	MTR			CYP2C38		H3F3A
	MYO5A			CYP2C39		HIST1H1A
	MYO5C			CYP2C55		HIST1H1B
	MYT1L			CYP3A25		HIST1H1C
	NAA25			CYP3A41A		HIST1H1E
	NAPA			CYP3A44		HIST1H3A
	NAV2			CYP3A57		HIST1H3B
	NBAS			CYP3A59		HMGCS1
	NDRG1			DCTN1		HMGCS2
	NOS3			DECR2		HOPX
	NT5DC1			DERL1		HPGD
	NUDT2			DGKE		HSCB
	NUTF2			DHCR24		IGSF5
	PACS2			DHX37		IL1RAP
	PAPSS2			DMPK		IREB2
	PARP4			DNAH7B		ITGA2B
	PBDC1			DNAJA2		KANSL3
	PEBP1			DNAJB2		KDSR
	PEX1			DUSP3		MAN2C1
	PEX12			DYNC1LI2		MAT1A
	PEX5			ECHDC1		ME1
	PHYKPL			EEPD1		MGLL
	PI4KA			ENTPD5		MGME1
	PIR			EPHX1		MGMT
	PLIN3			ERMP1		MMRN1
	PON1			F10		MRPS12
	PPCDC			F12		MTERF2
	PPP2R1A			F2		MTG1
	PRDX3			FAM175B		NCEH1
	PRKACA			FBXW9		NEBL
	PRKRA			FDFT1		NKTR
	PRMT9			FHAD1		NPRL3
	PSMA7			FKBP1A		OSBPL3
	PSMB2			FMO9		PANK2
	PSMD12			FUT8		PCTP

INH only	RIF only	RIF/INH	INH and RIF only, but not RIF/INH	RIF only and RIF/INH, but not INH only	INH only and RIF/INH, but not RIF only	INH only, RIF only, and RIF/INH
	PSMG1			GALE		PDK1
	PSMG2			GALK1		PDK2
	PXMP2			GGCT		PEX11A
	PYROXD2			GM10639		PEX16
	R3HCC1			GNPAT		PEX2
	RAB18			GP5		PF4
	RAB3GAP2			GPX4		PGD
	REEP3			GPX7		PGS1
	RNF14			GSTA1		PIGX
	RRP1B			GSTA4		PLEK
	SDR39U1			GSTM1		PLEKHA1
	SERHL			GSTM2		PLIN2
	SHPK			GSTM3		PNPLA2
	SIRT2			GSTM4		PNPLA3
	SLC25A10			GSTM5		POMP
	SNAP25			GSTM6		PRKCA
	STARD10			GSTM7		PSMG3
	TBC1D24			GSTT2		PTK2B
	TBCD			GSTT3		PTS
	TGM2			H2-Q10		RAB30
	TM7SF2			HAUS3		RARRES1
	TMED3			HGFAC		RBM8
	TMEM135			HSD17B12		RBM8A
	TMEM223			HSD17B4		REEP6
	TMUB2			HSD17B7		RPIA
	TMX2			HSPB1		RPL14
	TPRG1L			HTATIP2		RPL22L1
	TSR2			ICA		RPL24
	TXN			IFI27L2B		RPL28
	UBA5			IFIT1		RPL35
	UBFD1			ITPKB		RPL4
	UBL4A			KIAA0100		RPL6
	VPS13D			KIF21A		RPL7
	VTA1			KLHDC10		RPL7A
	WBSCR27			KNG1		RPL8
	WWC1			LAMA1		RPS6KA1
	ZW10			LGALS1		RRP36
				LHPP		RSL1D1

INH only	RIF only	RIF/INH	INH and RIF only, but not RIF/INH	RIF only and RIF/INH, but not INH only	INH only and RIF/INH, but not RIF only	INH only, RIF only, and RIF/INH
				LPIN3		RTFDC1
				MAPRE3		SDC1
				MARCH6		SECISBP2L
				MASP2		SERPINB1A
				METTL7B		SERPINB1C
				METTL9		SERPINB6B
				MGST1		SLAIN2
				MICAL2		SLC47A1
				MSTO1		SLCO1A4
				MVP		SMTN
				MYO19		SNAP29
				MYO5B		SPP2
				N6AMT2		ST3GAL5
				NAA20		STS
				NADK		SVIP
				NAGK		SYNJ2
				NBEAL1		TDRKH
				NCF1		THBS1
				NEB		THRSP
				NMT2		TIMP3
				NOL3		TKT
				NQO1		TMCC3
				NRDE2		TMEM205
				NSDHL		TMEM263
				NUDT12		TMLHE
				OAT		TMPRSS11B
				OBFC1		TOP1MT
				ODR4		TPD52
				P4HTM		TRIAP1
				PAFAH2		TRUB2
				PDXP		TSSC4
				PEO1		TTR
				PEX10		UCK1
				PEX11G		USP40
				PEX13		USP45
				PEX14		VNN1
				PEX19		VNN3
				PEX26		WDR73

INH only	RIF only	RIF/INH	INH and RIF only, but not RIF/INH	RIF only and RIF/INH, but not INH only	INH only and RIF/INH, but not RIF only	INH only, RIF only, and RIF/INH
				PEX3		ZNF22
				PEX6		
				PGLYRP2		
				PGRMC2		
				PITRM1		
				PLA2G6		
				PLIN4		
				PLTP		
				PNPLA8		
				POLR1E		
				POR		
				PROZ		
				PRUNE		
				PSAT1		
				PSMD5		
				PUSL1		
				PXMP4		
				PZP		
				RBP4		
				RDH16		
				RDH9		
				REEP5		
				REXO2		
				RIN2		
				ROBO1		
				RPF2		
				RTP4		
				SAV1		
				SDHAF1		
				SERPINA6		
				SERPINA7		
				SERPINC1		
				SERPINF1		
				SERPINF2		
				SHCBP1L		
				SLC22A18		
				SLPI		
				SMAD2		

INH only	RIF only	RIF/INH	INH and RIF only, but not RIF/INH	RIF only and RIF/INH, but not INH only	INH only and RIF/INH, but not RIF only	INH only, RIF only, and RIF/INH
				SRXN1		
				STAU2		
				STRADB		
				SULT2A1		
				SYAP1		
				SYNPO		
				TBCE		
				TBCEL		
				TCN2		
				TDRD7		
				TEP1		
				TEX2		
				TMA7		
				TMEM109		
				TMEM120A		
				TMEM259		
				TMEM51		
				TMX1		
				TNFSF10		
				TRAK2		
				TTC7		
				TTPA		
				TXNDC15		
				TYW1		
				UAP1L1		
				UBASH3B		
				UBXN4		
				UCKL1		
				UGDH		
				UGT1A1		
				UGT1A10		
				UGT1A5		
				UGT1A7C		
				UGT1A9		
				UGT2B34		
				UGT2B35		
				UPP1		
				UROS		

INH only	RIF only	RIF/INH	INH and RIF only, but not RIF/INH	RIF only and RIF/INH, but not INH only	INH only and RIF/INH, but not RIF only	INH only, RIF only, and RIF/INH
				USE1		
				WDR34		
				WDR77		
				WWOX		
				ZBTB21		
				ZCCHC11		
				ZFPL1		
				ZNF804A		
				ZNHIT2		

INH, isoniazid; RIF, rifampicin; RIF/INH, rifampicin and isoniazid combination treatment.

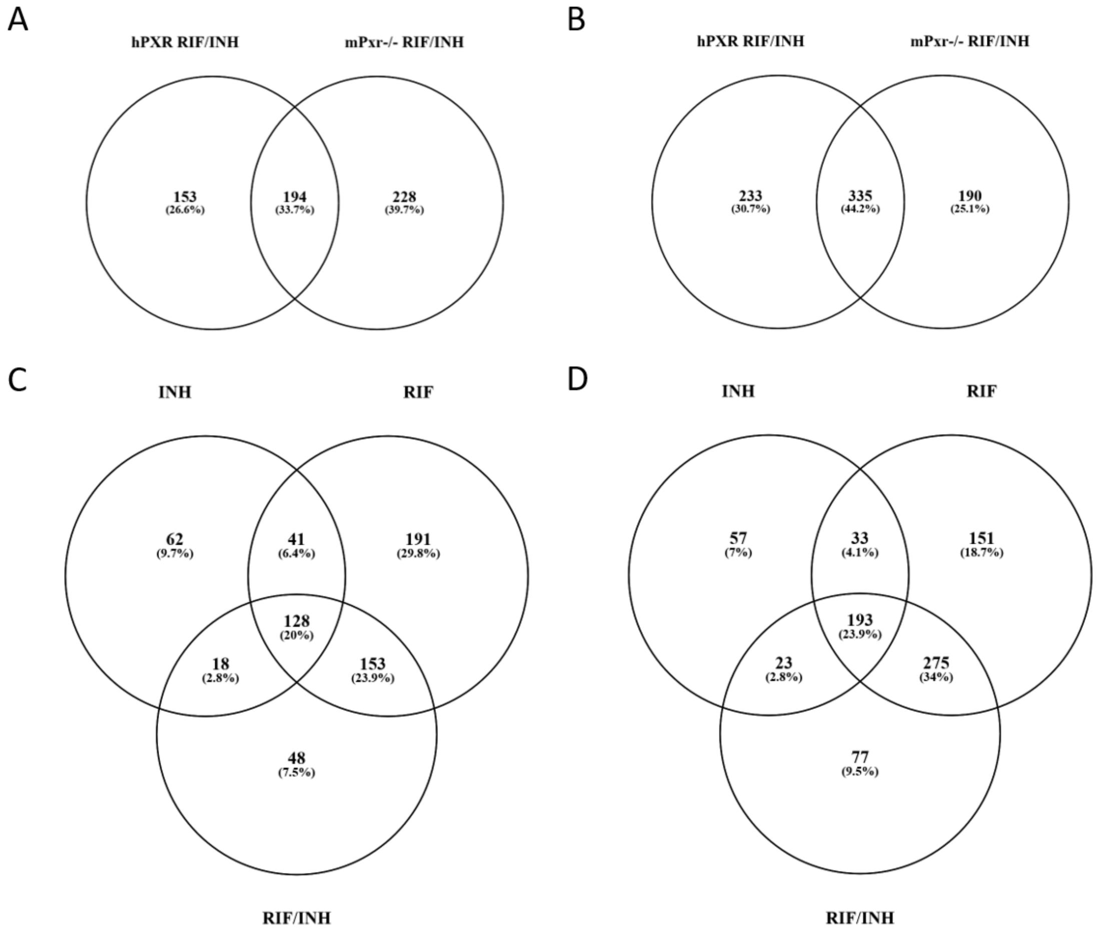

Supplementary Figure S1. Venn diagrams of protein expression changes. (**A**, **B**) Venn diagrams demonstrating the overlap between and specificity of proteins changed in the different mouse strains (*hPXR* or $mPxr^{-/-}$ mice) and (**C**, **D**) *hPXR* mice drug treatment groups. Upregulated proteins (B, D) and downregulated proteins (A, C) are shown. Only changes with a magnitude greater than 1.15-relative or less than 0.85-relative with a significance of $p < 0.05$ are displayed, as determined by one-way ANOVA with Dunnett post hoc analysis.

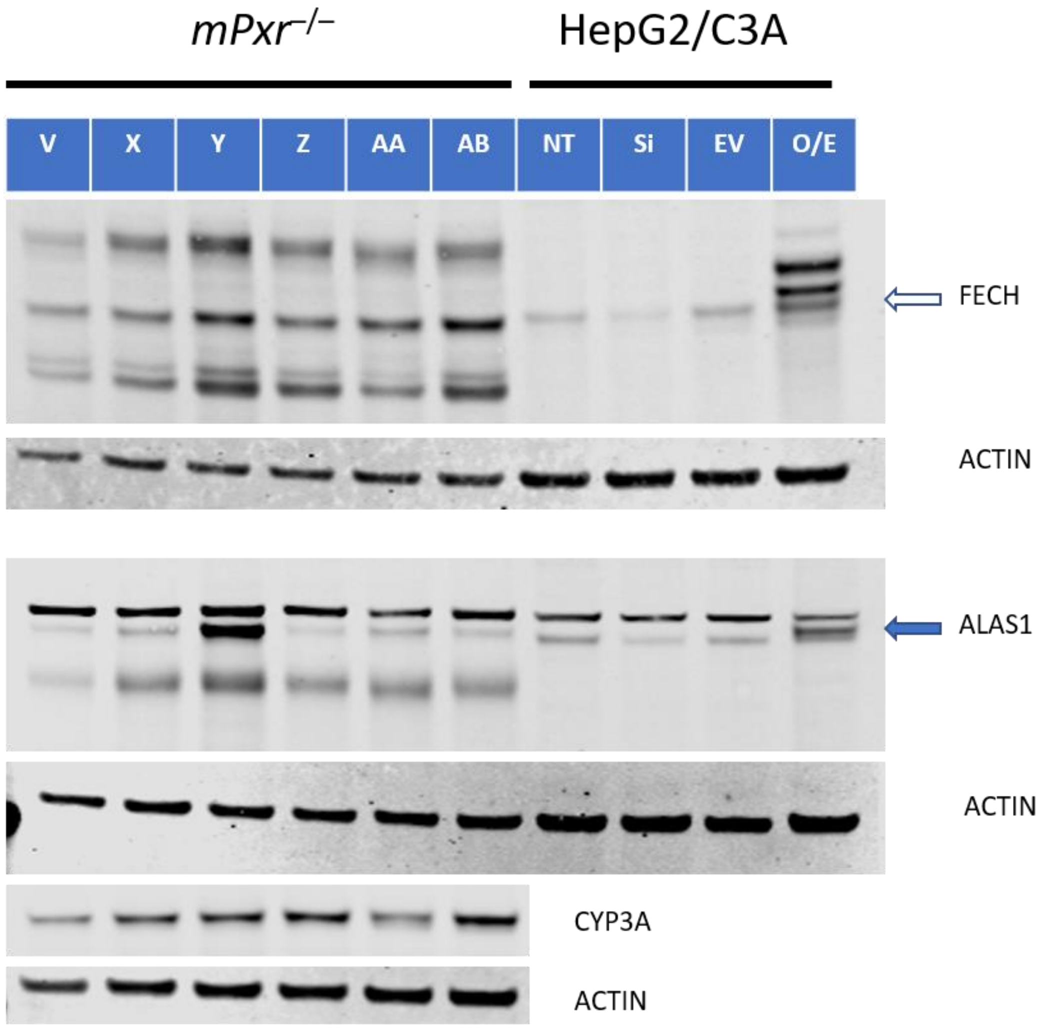

Supplementary Figure S2. Protein expression of mouse *mPxr*$^{-/-}$ liver samples considered for proteomics analysis. Immunoblotting of FECH, ALAS1, and CYP3A in *mPxr*$^{-/-}$ mouse livers for selection in proteomic analysis (lanes 1-6). For HepG2/C3A (lanes 7-10): NT, non-targeting transfected control siRNA3 (Cat # D-001210-03); Si, either HepG2/C3A lysate siFECH (Cat #D-011036-01-0002) (top two panels), or siALAS1 (Cat # M-009276-00) (bottom two panels); EV, empty vector (pcDNA3); O/E, ectopic overexpression of FECH (top two panels) or ALAS1 (bottom two panels) proteins from plasmids. Letters under treatment groups indicate individual mice. Closed arrow indicates mature ALAS1. Open arrow indicates FECH.

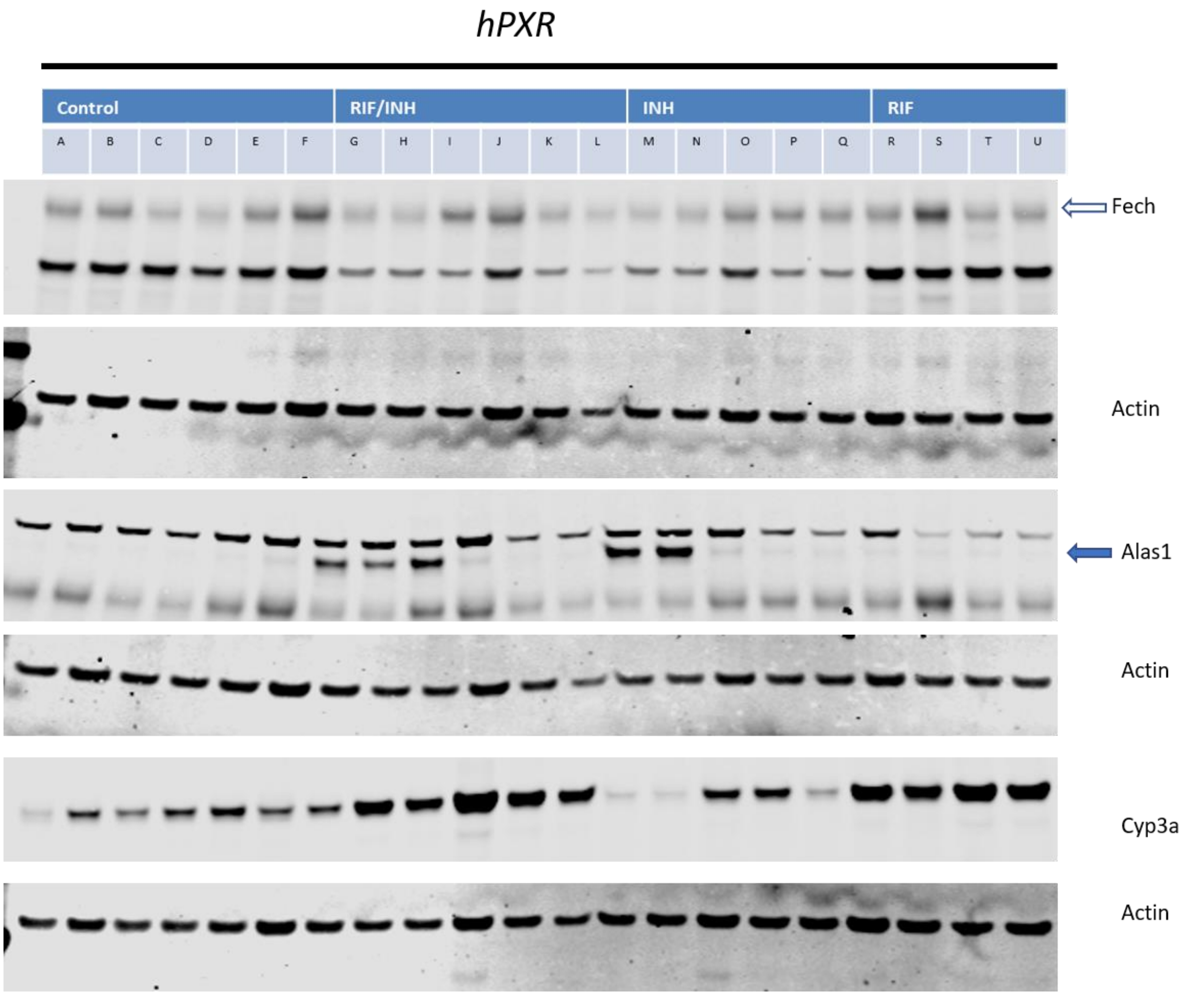

Supplementary Figure S3. Protein expression in mouse *hPXR* liver samples considered for proteomics analysis. Immunoblotting of FECH, ALAS1, and CYP3A in *hPXR* mouse livers for selection in proteomic analysis. Letters under treatment groups indicate individual mice. Closed arrow indicates mature ALAS1. Open arrow indicates FECH.

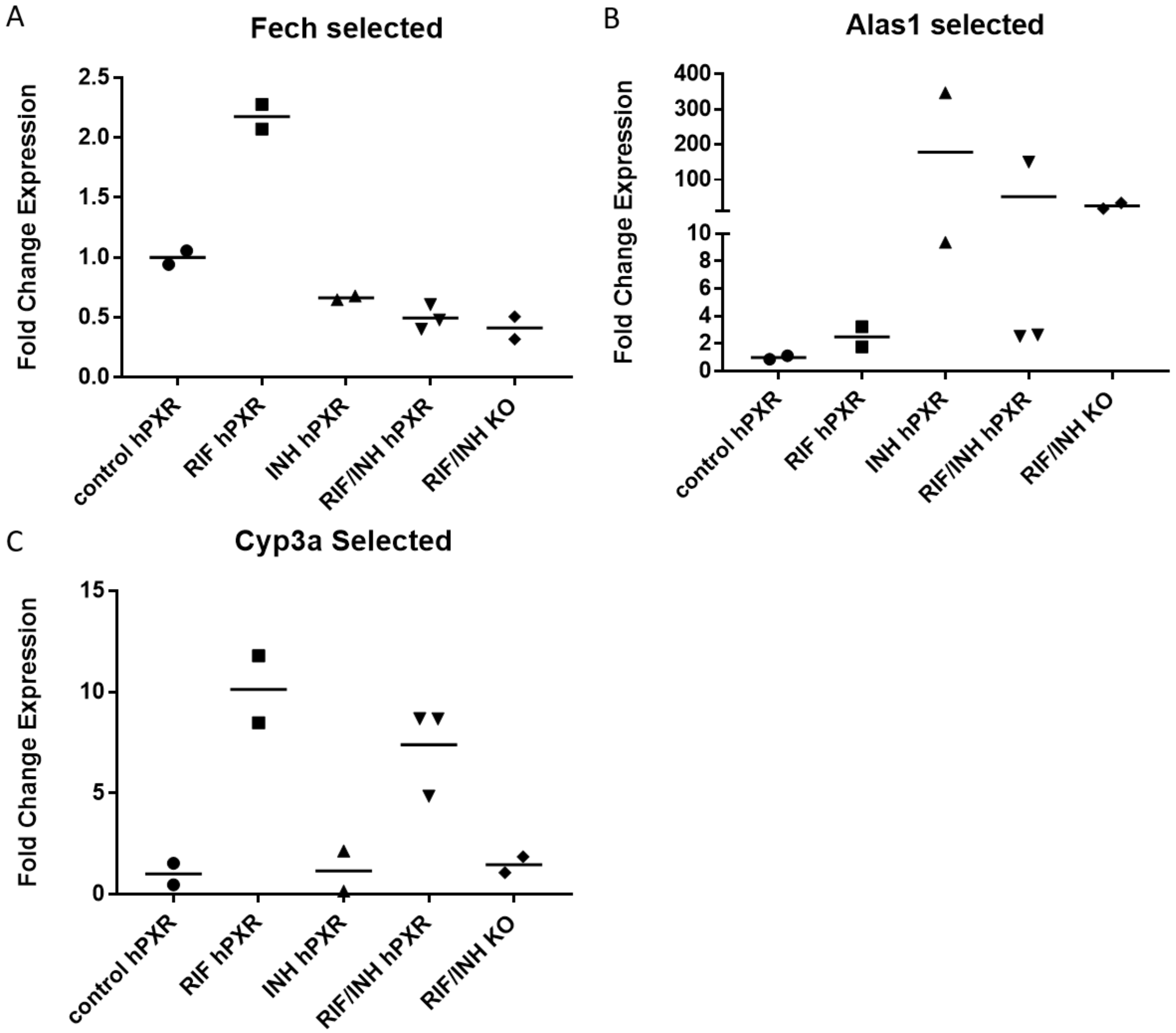

Supplementary Figure S4. Quantification of Western blots of mouse livers selected for proteomics analysis. (**A**) FECH, (**B**) ALAS1, and (**C**) CYP3A.

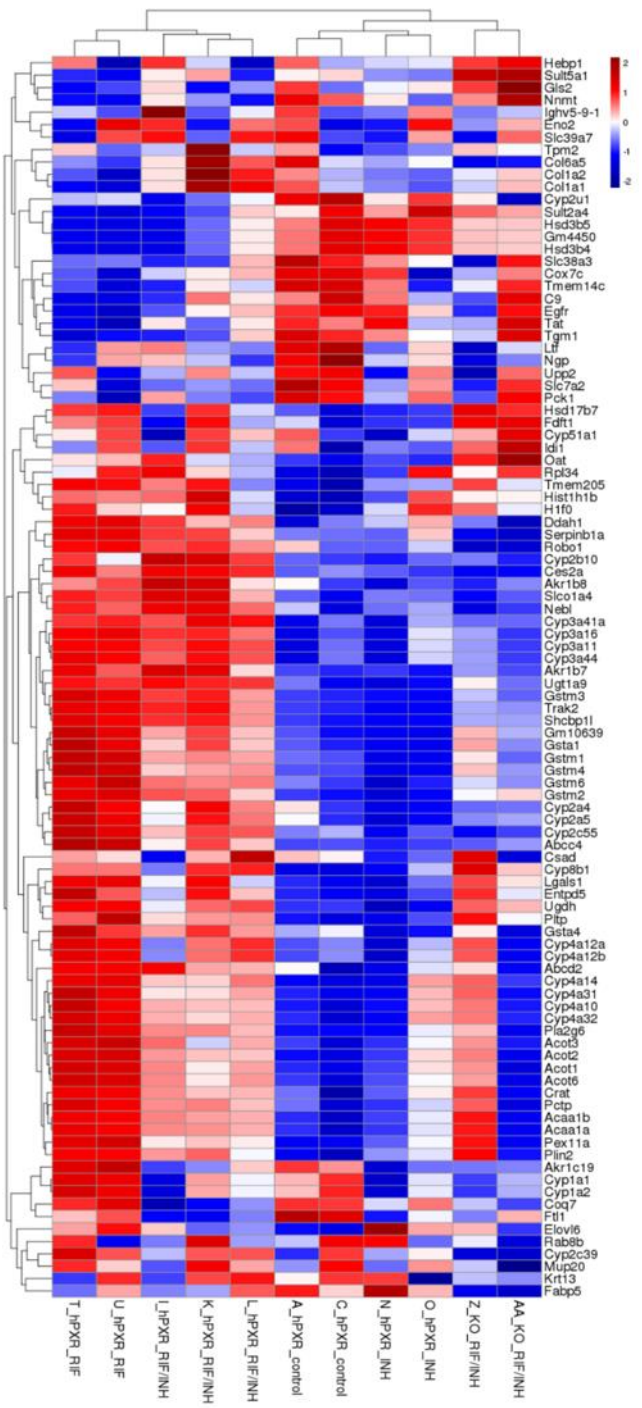

Supplementary Figure S5. Hierarchical clustering analysis for mouse liver proteomic profiling. Hierarchical clustering of the top 100 most variable differentially expressed proteins among the treatment groups. Low-expression proteins (peptide sequence matches < 10) were filtered, and the top 100 most variable proteins are displayed. Only changes with $p < 0.01$ are displayed (one-way ANOVA with Dunnett post-hoc analysis). Z-scores are shown, in which red indicates upregulated and blue indicates downregulated proteins. Letters (T, U, I, K, L, A, C, N, O, Z, AA) indicate individual mice. hPXR_INH = *hPXR* mice treated with isoniazid; hPXR_RIF = *hPXR* mice treated with rifampicin; KO_RIF/INH = $mPxr^{-/-}$ mice treated with rifampicin and

isoniazid; hPXR_RIF/INH = *hPXR* mice treated with both rifampicin and isoniazid. hPXR_control = *hPXR* mice treated with vehicle.

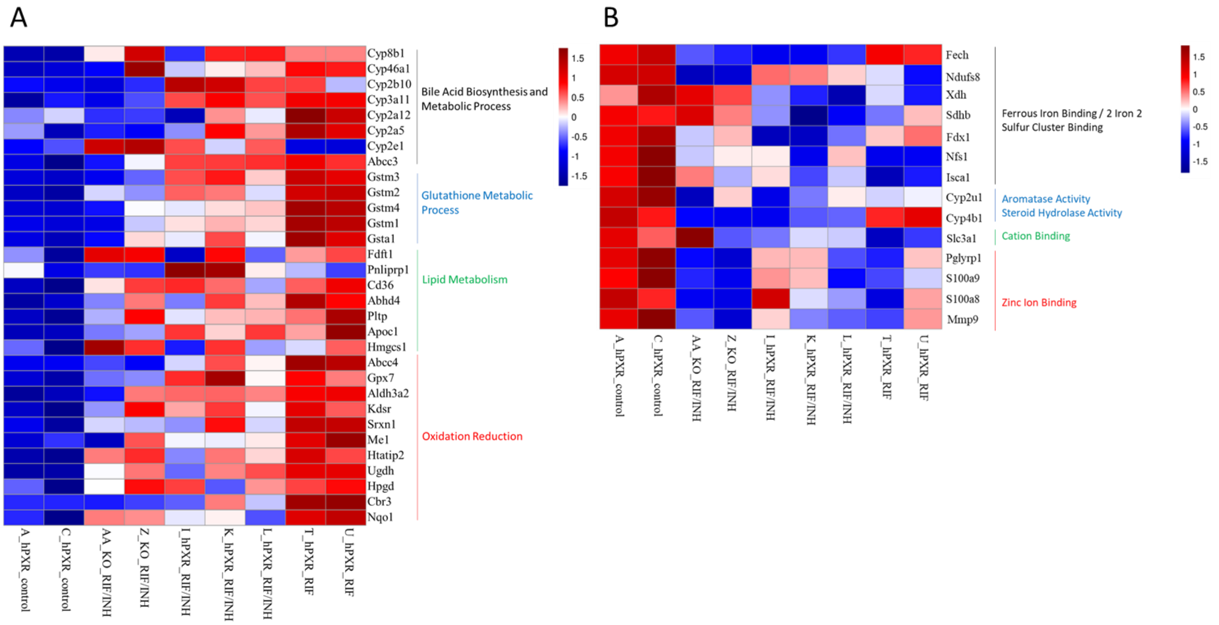

Supplementary Figure S6. Heatmap of selected protein changes. Z-scores are shown, in which red indicates upregulated and blue indicates downregulated proteins. Letters (T, U, I, K, L, A, C, Z, AA) indicate individual mice. hPXR_RIF = *hPXR* mice treated with rifampicin; KO_RIF/INH = *mPxr*$^{-/-}$ mice treated with rifampicin and isoniazid; hPXR_RIF/INH = *hPXR* mice treated with both rifampicin and isoniazid. hPXR_control = *hPXR* mice treated with vehicle.
